# Development, validation, and implementation of the antibody-secreting cell maturity index: Universal prediction of human plasma cell maturity

**DOI:** 10.1016/j.isci.2026.116050

**Published:** 2026-05-22

**Authors:** Tobit D. Steinmetz, Guiyou Yang, Tessa de Nooijer, Nisha van der Meer, Naomi Karmi, Rick Wilbrink, Hendrika Bootsma, Gwenny M. Verstappen, Frans G.M. Kroese

**Affiliations:** 1 University of Groningen, University Medical Center Groningen, Department of Rheumatology and Clinical Immunology, 9700RB Groningen, the Netherlands; 2 University of Groningen, University Medical Center Groningen, Department of Epidemiology, 9700RB Groningen, the Netherlands; 3 University of Groningen, University Medical Center Groningen, Department of Oncology, 9700RB Groningen, the Netherlands; 4 University of Groningen, University Medical Center Groningen, Department of Gastroenterology and Hepatology, 9700RB Groningen, the Netherlands

**Keywords:** Artificial intelligence, Components of the immune system

## Abstract

Defining the maturity of long-lived antibody-secreting cells (ASCs) is important for vaccine optimization and research into autoimmune diseases, but current assessment of plasma cell maturity is limited. We developed a universal, robust method to define plasma cell maturity using a meta-analysis of public, human cytometry data, with harmonized expression of ASC maturity markers across samples. Vaccination or infection samples were selected to train a random forest-based machine learning prediction model (performance of r = 0.866). After evaluation (performance of r = 0.616), the final model, named the antibody-secreting cell maturity index (ASC-ME), was applied to compartments with long-term ASC survival niches, namely bone marrow and gut. Both compartments showed increased ASC maturity, biologically validating the ASC-ME model. Example analyses highlight the broad spectrum for model application, e.g., in vaccine research, clinical trials or in ASC-related autoimmune diseases. Overall, our ASC-ME model, accessible via an online platform, offers a robust approach in evaluating ASC maturity.

## Highlights

• An algorithm trained on immune reaction kinetics predicts ASC maturity
• The ASC-ME model integrates eight cell markers into a continuous maturity readout
• Application of the ASC-ME model is in line with current knowledge of ASC biology
• The ASC-ME model is openly accessible through an online tool

## Graphical abstract

**Figure:**
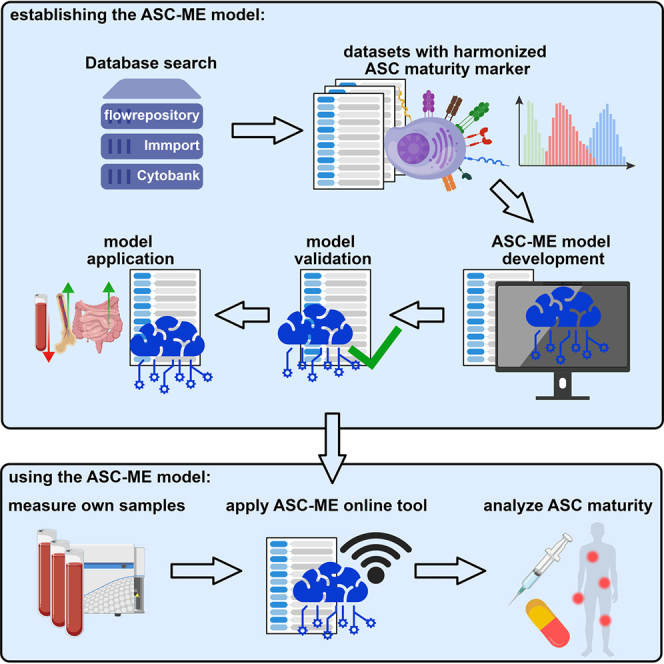

## Introduction

Persistent antibody production by plasma cells is a double-edged sword of the immune system. Vaccination or infection can induce protective antibodies that maintain stable titers in the blood for decades.^1^ On the other hand, harmful autoantibodies can be present for years prior to the diagnosis of several autoimmune diseases,^2^^,^^3^ possibly contributing to disease pathogenesis.^4^^,^^5^ Stable antibody titers are upheld by long-lived plasma cells that presumably have lifespans similar to their host^6^ and often reside in the gut^6^ or bone marrow.^7^^,^^8^

Activation of B cells triggers differentiation into plasmablasts and plasma cells, together referred to as antibody-secreting cells (ASCs) (reviewed in Gaudette and Allman^9^ and Steinmetz et al.^10^). Newly formed immature ASCs (plasmablasts) still proliferate and phenotypically resemble activated B cells.^11^ The continuous maturation process from immature ASCs toward fully mature ASCs (plasma cells) includes phenotypical changes and homing of ASCs to long-term survival niches^12^^,^^13^ in bone marrow^14^^,^^15^ and gut.^6^^,^^16^ In human samples, CD27 and CD38 are well-established ASC identifiers in flow and mass cytometry. Immature ASCs typically express cellular markers associated with activated B cells, such as CD19, CD20, CD45, CD69, CD86, Ki67, and HLA-DR that decrease during ASC maturation. Other markers such as CD28, CD44, CD56, CD138, BCMA, TACI, and BCL2 are upregulated during ASC maturation. The up- and downregulation of these markers presumably follows individual kinetics.^6^^,^^7^^,^^8^^,^^11^^,^^17^^,^^18^

In general, immune reactions generate a wave of new, immature ASCs. Most of these cells die within a few days or weeks, called short-lived ASCs. Only a minority of immature ASCs transition into the long-lived ASC pool and fully mature.^19^ This maturation is influenced by ASC intrinsic properties, the ASC survival niche, or a combination of both.^20^^,^^21^ Consequently, the blood ASC pool primarily consists of newly formed ASCs, with only a minor fraction of mature, long-lived ASCs leaving survival niches.^22^ Even so, blood is an easy-accessible and important source of ASCs in human immunology.

Maturity and longevity including their cellular markers are not fully defined concepts in ASC biology. ASC maturation is slow, and owing to this, only ASCs with long lifespans achieve full maturity. Consequently, long-lived ASCs and mature ASCs are considered largely overlapping populations.^11^ Studies investigating long-lived ASCs often rely on defining these cells as CD19^−^CD138^+^^8^^,^^23^ or CD19^−^CD45^−6^, but analysis of antigen-specific ASCs from decade-old immune reactions revealed that long-lived ASCs can express CD19 or lack CD138.^24^^,^^25^ To define mature ASCs more reliably, we previously created an ASC maturity indexing approach scoring CD19, CD28, CD138, and HLA-DR expression by ASCs.^26^ However, this approach lacked comparability between datasets, standardization, and validation using samples of controlled immune reactions.

A robust method to assess the maturity of ASCs permits evaluation of the ability of vaccines to induce mature ASCs, critical for the upkeep of protective antibody titers. Identifying the maturity state of ASCs in autoimmune diseases may lead to better disease monitoring, enable better patient stratification or identification and facilitate personalization of treatment strategies. Given these benefits of a generalizable tool to assess ASC maturity, we utilized a large collection of human cytometry data from timed vaccination and controlled infection studies. Leveraging these data, we trained a machine learning algorithm to create and evaluate a maturity prediction model, termed the antibody-secreting cell maturity index (ASC-ME) model. Using several clinically relevant examples, we highlight potential areas of application for our ASC-ME model.

## Results

### Meta-analysis and harmonization of publicly available cytometry samples

**Figure 1:**
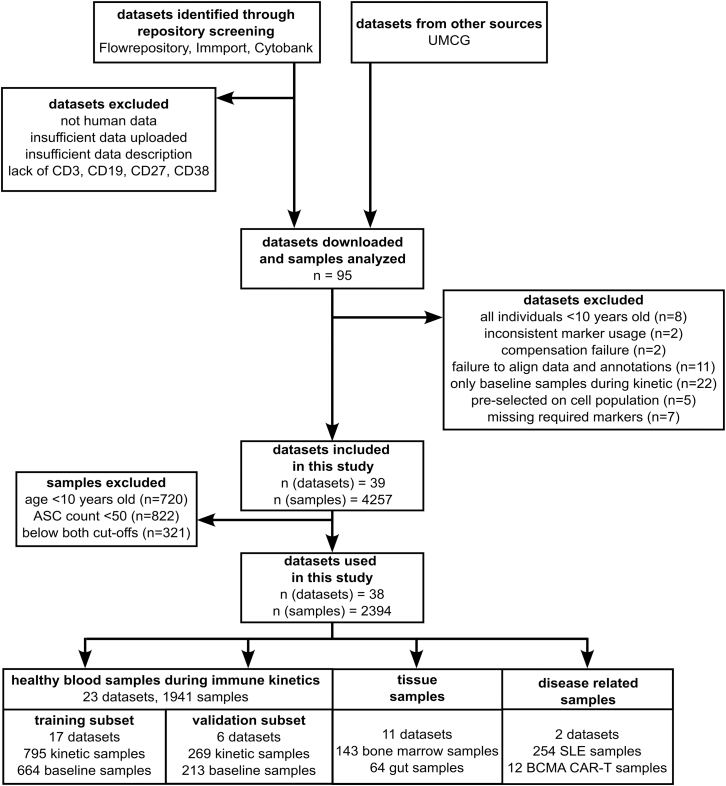
Flow diagram of the design of the ASC-ME study including sample collection, cleaning and distribution into the separate study subsets See also Figures S1, S2 and Table S2.

Mass and flow cytometry samples from 39 datasets comprising 4,257 human samples, stained with T cell and B cell markers required for ASC identification were included. Total blood ASCs were defined as live, single CD3^−^CD27^+^CD38^+^ cells (Figure S1). Only samples with an absolute ASC count ≥50 were included (Figure 1).

All healthy blood datasets were allocated either to training or validation data subsets, maintaining a ratio of approximately 75% training and 25% validation data regarding the numbers of samples (Figure 1). Within the training and validation datasets, female and male sex as well as ethnicity were similarly distributed (Figure S2).

### Changes of potential ASC maturity markers during immune reactions and with age

**Figure 2:**
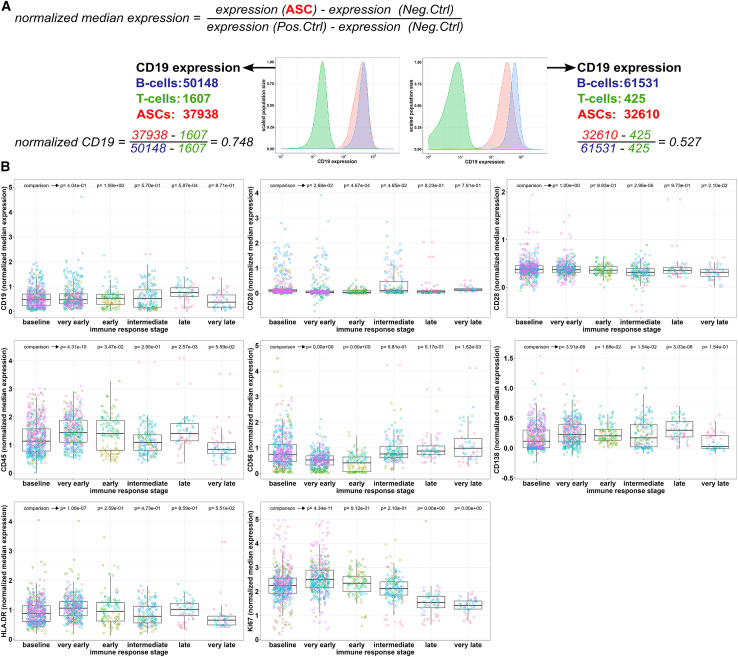
Normalization of maturity markers and expression during immune reactions (A) general formula to calculate normalized median expression and two examples with overlay histograms of CD19 expression by ASCs and control populations in order to apply the formula to calculate the normalized median expression of CD19. (B) Normalized median expression of CD19, CD20, CD28, CD45, CD56, CD138, HLA-DR, and Ki67 by total blood antibody-secreting cells during a timed immune reaction of 1459 longitudinal samples. Missing data were imputed. Data points colored according to individual datasets and boxplots represented as median with 95% confidence intervals. *p* Values calculated using one-way ANOVA with Tukey correction in comparison to baseline. See also Figures S1, S3, S4, S5 and Table S1.

Initially, a total of 14 potential maturity markers were included and normalized expression was calculated (Figure 2A). Six markers were omitted from further analysis due to specific limitations: low abundance within the datasets (CD44, BCL-2, BCMA, and TACI), batch effects (CD69), or lack of predictive value (CD86). The analysis was continued with the residual eight cellular markers: CD19, CD20, CD28, CD45, CD56, CD138, HLA-DR, and Ki67 (Table S1; Figure 2; Figure S1B). Marker changes over time were assessed within the training samples during ongoing immune responses with the day post-immune-intervention (DPI) time point DPI ≤1 as baseline. *De-novo* generation of immature ASCs starts within the first week of the immune response, presumably lowering the average ASC maturity in the blood. Baseline and kinetic samples from the 17 training datasets (see Figure 1, in total 1,459 samples) were grouped by immune response stages into baseline (DPI: ≤1), very early (DPI: 5–10), early (DPI: 10–19), intermediate (DPI: 20–50), late (DPI: 51–99), and very late immune responses (DPI: 100–180). With this approach, we observed unique expression patterns for each of the selected markers during the immune response. Median expression of CD19, CD45, and HLA-DR by circulating ASCs temporarily increased in comparison to baseline at different stages during the immune response. In contrast, median CD56 expression temporarily decreased in the very early and early immune stages compared to baseline and was elevated at the very late immune stage (Figure 2B). Expression of CD138 and Ki67 first increased at the very early stage followed by a drop in expression during the consecutive stages, especially for Ki67. No changes in expression of CD20 and CD28 during immune responses were found (Figure 2B). In general, expression patterns for all eight markers were homogenous across all training and validation data (Figure S3).

Since ASC maturity presumably increases over a lifetime, normalized expression of maturity markers was analyzed in the context of the individuals’ age only in unvaccinated samples, i.e., baseline DPI (17 datasets, 664 samples). CD45, CD56, and CD138 expression by ASCs positively correlated with age, albeit, with poor to fair correlation coefficients of r = 0.33, r = 0.28 and r = 0.43, respectively (Figure S4). CD19 and CD20 expression negatively correlated with age; however, data points are only elevated between 10 and 20 years of age. No correlation of age with the other markers (CD28, HLA-DR, and Ki67) was observed (Figure S4). When assessing the eight potential maturity markers prior to data imputation, similar increasing and decreasing expression patterns were observed in comparison to imputed data both during the immune response stages (Figure S5A) and with the age of individuals (Figure S5B).

### A random forest-based prediction model for blood ASC maturity in healthy individuals

Complementary to immune stage groups, we also assessed correlations of the potential maturity markers with DPI. Only Ki67 and CD56 showed fair correlations with DPI with correlation coefficients of r = −0.490 and r = 0.397, respectively (Figure S6). Given this low predictive capacity of single markers and our observations that some markers depict transient changes or plateau at different immune stages, we next attempted to increase the predictive capabilities of ASC maturity markers via a machine learning method. To this end, we trained a random forest model incorporating eight ASC maturity markers (Table S1) with the assumption that the predicted DPI time point is a suitable surrogate for ASC maturity. As outcome, we aimed for a method that predicts the DPI for samples of unknown immune state and, therefore, their ASC maturity.

**Figure 3:**
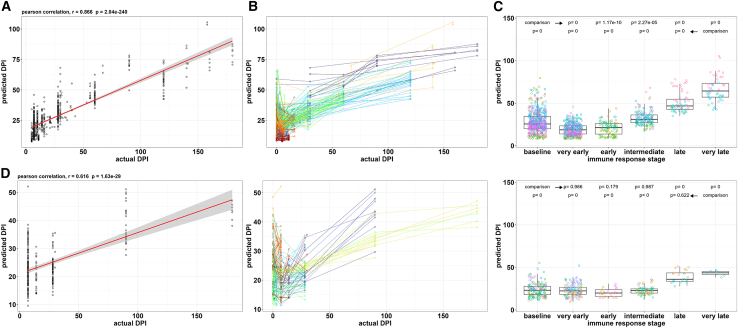
Establishing a random forest-based maturity prediction model for antibody-secreting cells using training data from healthy individuals (A) Pearson correlation of actual day post-immune-intervention (DPI) and predicated DPI with linear regression and 95% confidence intervals (red line with gray area). (B) Longitudinal data of timed immune reactions including baseline samples; data points from the same individuals are connected. (C) Training data grouped by immune response stages including baseline samples. Data points colored according to individual datasets and boxplots represented as median with 95% confidence intervals. *p* Values calculated using one-way ANOVA with Tukey correction in comparison to baseline and very late immune stage. (D) Analog assessment as in (A)–(C) for validation data. See also Figures S6, S7 and S8.

The random forest prediction model was created using training data from very early to very late immune response stages of 795 available kinetic samples from 17 datasets previously analyzed for individual markers aforementioned (see STAR Methods: Setup for the maturity prediction model using random forest, or Supplemental Appendix for more details). The final model was named ASC-ME and evaluated by correlating actual DPI and predicted DPI yielding a very strong Pearson coefficient of r = 0.866 (Figure 3A), with predicted DPI as indicator of ASC maturity. Evaluating multiple time points from the same individuals and including baseline samples showed a drop in predicted DPI for most samples early in the immune reaction followed by a gradual increase in predicted DPI (Figure 3B). This pattern was also observed when evaluating the respective studies separately (Figure S7). This initial drop in DPI is in line with the generation of new, immature ASCs starting around one week after an immune response.^27^ When grouped into immune response stages (very early to very late, as described previously), a decrease in predicted DPI during the initial, very early and early responses was followed by a stepwise significant increase in predicted DPI (Figure 3C).

External validation of the ASC-ME model using 269 samples from six datasets that were not used for model training yielded a moderate Pearson correlation coefficient of r = 0.616 for predicted DPI and actual DPI with similar observations for longitudinal data from the same individuals and DPI groups (Figure 3D).

To evaluate the influence of the ASC count cut-off on the model performance during training and validation, similar models were generated that included either all samples with ASC counts, ≥10, or ≥100 in comparison to the previously established model with an ASC count ≥50. While performance of model training was comparable, the correlation coefficient during validation decreased with the number of available samples within the validation subset dependent on the respective cut-off (Figure S8A). The ASC count ≥50 cut-off was chosen for the final model as a suitable balance between reliable readout of median fluorescence intensity (expression) values from ≥50 ASCs per sample and available sample numbers. To evaluate whether the large differences in sample size among the studies used are influencing the model development, we randomly down-sampled the training and validation datasets to equal samples number per study. Since down-sampling did not improve the model performance (Figure S8B), we continued with the model of the full datasets.

### Evaluation and characteristics of the ASC-ME prediction model

**Figure 4:**
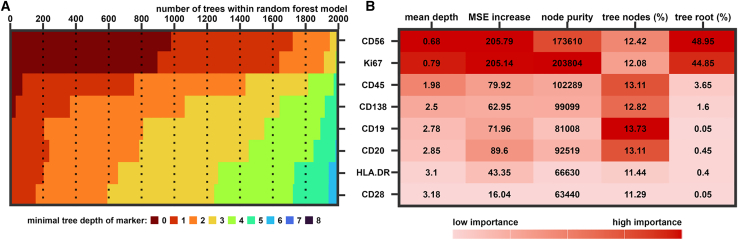
Relevance of individual prediction markers to the ASC-ME model (A and B) Heatmaps of (A) minimal tree depth for each maturity marker used as predictor within the random forest model (depth 7 and 8 not visible due to small numbers) and (B) various importance parameters; red-scale according to highest (dark red) and lowest (light red) importance of each parameter.

In order to evaluate the contribution of the eight individual maturity markers to the final ASC-ME model, the minimal tree depth of each marker was determined (Figure 4A). Parameters at the root of the tree or at low depth have a higher impact on the tree outcome. The markers CD56 and Ki67 were used in most trees (48.95% and 44.85% of 2,000 trees, respectively) as root of the tree (depth = 0) and showed the lowest mean depth of all markers followed by CD45 and CD138. However, all maturity markers were used with similar frequency among all depths of the tree structures ranging from 11.29%–13.73% of all tree nodes. Mean squared error (MSE) increase, indicating loss in model performance if a particular marker is randomized, was highest for CD56, Ki67, CD20, and CD45, respectively (Figure 4B).

### Application of the maturity prediction model to various clinical settings

**Figure 5:**
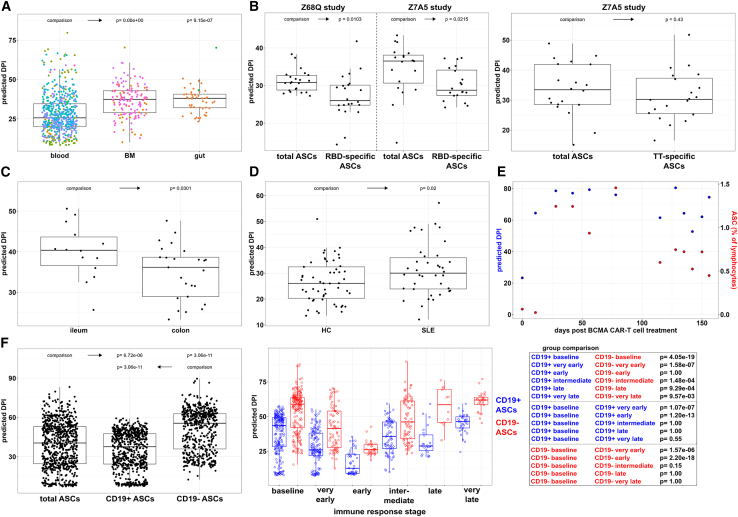
Various ways of applying the maturity prediction model (A) Predicted day post-immune-intervention (DPI) in blood, bone marrow (BM), and gut samples. Data points colored according to individual datasets and boxplots represented as median with 95% confidence intervals. *p* Values calculated using one-way ANOVA with Tukey correction in comparison to blood samples. (B and C) DPI prediction (B) in two BM datasets after SARS-CoV2 vaccination for total ASCs, for receptor binding domain (RBD)-specific and tetanus toxoid (TT)-specific ASCs and (C) in gut samples by location. Data represented as boxplots with median and 95% confidence intervals. *p* Values calculated using Mann-Whitney U test. (D) Comparison of DPI prediction for healthy controls (HC) and patients with systemic lupus erythematosus (SLE) at the inclusion time point of the respective study. Data represented as boxplots with median and 95% confidence intervals. *p* Value calculated using students t-test. (E) Predicted DPI during follow-up in a case report of B cell maturation antigen (BCMA) chimeric antigen-receptor (CAR)-T cell treatment (left *y* axis) in relation to the frequency of blood ASC as percentage of total lymphocytes (right *y* axis). (F) Assessment of CD19^−^ and CD19^+^ ASC subpopulations at all time points in comparison to total ASCs, represented as boxplots with median and 95% confidence intervals using one-way ANOVA with Tukey correction (left). CD19^−^ and CD19^+^ ASC subpopulations during ongoing immune reactions (middle), represented as boxplots with median and 95% confidence intervals compared with a Kruskal-Wallis test with Bonferroni correction (right). See also Figures S9 and S10.

After training and validating the ASC-ME model to describe maturity changes during controlled settings like timed infections and vaccinations, we assumed that the ASC-ME model can translate the maturity marker phenotype of ASCs into a maturity prediction. Consequently, we next applied the ASC-ME model to various clinical settings to assess the ASC maturity independent of a known immune reaction. Predicted DPIs, generated using the ASC-ME model, were significantly higher in bone marrow and gut compared to blood samples, reflecting increased average ASC maturity in these tissues (Figure 5A). In a subset of bone marrow samples,^28^ ASCs were identified antigen-specifically for the SARS-CoV2 receptor-binding domain (RBD) and for tetanus-toxoid (TT). RBD-specific ASCs acquired from the bone marrow 25–312 days after CoV2-vaccination, exhibited significantly lower predicted DPI in two independent datasets,^28^^,^^29^ while TT-specific ASCs from one dataset showed no significant reduction (Figure 5B). Further analysis of gut samples^30^ revealed a lower predicted DPI in colon ASCs than ileum ASCs (Figure 5C). The ASC-ME model was also applied to a study^31^ with systemic lupus erythematosus (SLE) patients. At baseline, predicted DPI values showed a significant increase in SLE compared to healthy individuals (Figure 5D). Blood samples from a multiple myeloma patient^32^ receiving B cell maturation antigen (BCMA) chimeric antigen receptor (CAR)-T cell treatment were analyzed using the ASC-ME model. A marked increase in predicted DPI was observed following treatment administration at the earliest follow-up time point on day 11 with persistently high predicted DPI during the subsequent study period accompanied by a similar pattern of increased frequency of blood ASCs during the follow-up (Figure 5E). In order to investigate if CD19^+^ and CD19^−^ ASCs have different maturity characteristics, we extracted the expression values of all eight maturity markers for CD19^+^ and CD19^−^ ASC subpopulations separately. At baseline, CD19^−^ ASCs indeed showed a higher ASC maturity based on elevated predicted DPI compared to all ASCs and CD19^+^ ASCs. However, during an ongoing immune reaction, also CD19^−^ ASCs showed decreasing predicted DPI values particularly at the early immune stage time point (Figure 5F). This could indicate that the CD19^−^ ASC subpopulation also contains immature ASCs. A comparable decrease during early immune reactions could not be observed for Ki67^-^ ASCs (Figure S9). An alternative prediction model without Ki67 as predictor showed very similar overall outcomes but performed only with a fair correlation during model validation (Figure S10).

## Discussion

We propose a universal ASC maturity prediction tool: the ASC-ME, to overcome the current lack of standardized ASC maturity assessment. In a meta-analysis of flow and mass cytometry data, expression of 14 potential ASC maturity markers was quantified in human blood samples with timed vaccination or controlled infection. These experimental conditions uniquely allowed for the assessment of the ASC maturation kinetic, as new immune reactions generate immature ASCs that mature over time.

The observed decrease in CD56 and increase in CD45, HLA-DR, and Ki67 expression after the influx of new, immature ASCs into the blood (as summarized in very early and early immune stage time points) is in line with previous reports. However, the increase in CD138 expression at the very early and early time points is unexpected and requires further investigation. With regard to predicted ASC maturity in late and very late immune stages, we also observed an unexpected increase of ASC maturity above the baseline level, instead of reaching a plateau similar to baseline samples which reflect the steady-state condition. Since some studies with long follow-up durations were vaccination studies that excluded participants with infections occurring during the follow-up time, the samples allocated to the late and very late time points likely reflect individuals that experienced a longer infection-free period than individuals at baseline.

The trained prediction model based on eight maturity markers provided a very strong correlation of actual DPI and predicted DPI (r = 0.866), with a moderate correlation during external validation (r = 0.616). A general high variance of predicted DPI values at any given actual DPI was observed on overall population level. When individuals were followed longitudinally, an overwhelming majority of samples showed an initial drop in predicted maturity, as mirrored by DPI values, followed by a consecutive increase over time. This highly variable maturity at baseline in combination with very similar maturity kinetics might originate from varying factors on the individual level like pre-existing immunity against the respective antigen and the corresponding antibodies or memory B cells. Other factors that vary the onset of ASC generation are antigen retention and time of antigen availability after vaccination and infection, the activation of B cells via the extrafollicular or the germinal center response and the number of cycles that B cells undergo within the germinal center reaction. However, the observed persistent maturity kinetics following single subjects could indicate that the ASC-ME model is suitable for describing the generation of new ASCs (as this initial drop in maturity) followed by ASC maturation over time at least on the individual level. Application of the ASC-ME model to a case of BCMA CAR-T cell treatment further underlines this aspect by demonstrating low variance of the ASC-ME model prediction in multiple repeated samples within a narrow time frame.

The predicted DPI as outcome of the ASC-ME model is reliably increased in samples with assumed high ASC maturity such as at late immune response stages and in tissue samples. Additionally, our observation of increased ASC maturity in untreated SLE patients is a confirmation of previous observations and likely reflects a disease-dependent increase in maturation in combination with resistance to apoptosis specific for SLE ASCs.^33^ Based on our results, we interpret the predicted DPI as outcome of the ASC-ME model as reflection of the average ASC maturity of a sample of an unknown immune stage. Consequently, the predicted DPI could be interpreted as surrogate for ASC maturity.

Analysis of the ASC-ME model characteristics revealed CD56 and Ki67 as the most important predictors for ASC maturity with highest tree root percentage, resembling a high impact of CD56 and Ki67 expression on the outcome of those decision trees. Additionally, both markers showed the highest MSE increase (i.e., permutation importance), which indicates the loss in model performance if this marker is removed from the model. However, correlating only CD56 or Ki67 expression with the actual DPI yielded lower Pearson coefficients (r = 0.397 or r = −0.490, respectively) than for the predicted DPI. This argues for an added value by combining various maturity markers as in the ASC-ME model. According to MSE increase, this added predictive value originates from the following markers with decreasing importance: CD20>CD45>CD19>CD138. Consequently, it might be beneficial to measure the expression of the relevant markers, especially CD56 and Ki67, directly, instead of relying on data imputation for optimal use of the ASC-ME model.

Our ASC-ME prediction model could be used as alternative approach that avoids previous limitations in assessing ASC maturity by integrating the expression of eight distinct maturity markers into a single, quantifiable maturity value (predicted DPI or ASC maturity, respectively). We utilized a high number of cytometry samples with broad diversity in sex, race, age, collection location, and combinations of assessed markers. The ASC-ME model demonstrated good performance during both training and validation phases. Biological validation was achieved by showing increased ASC maturity in bone marrow and gut tissue, as predicted by the ASC-ME model. Potential application of the ASC-ME model includes but is not limited to maturity assessment of vaccine-specific ASCs at different time points after vaccination to optimize vaccine components like adjuvants for early induction of mature ASCs. Furthermore, since maturation and IgG secretion are linked^34^ the ASC-ME model might assist in identifying ASCs with high secretion rates in various applications.

In summary, the ASC-ME model represents a suitable tool with the potential to be applicable across a broad range of clinical settings across immunology, oncology, and rheumatology.

### Limitations of the study

Limitations of this study result from extracting cytometry-based expression values from studies with vastly different standard operating procedures, including reagents, incubation protocols, and cell analyzers used. To mitigate batch effects resulting from these variations, data standardization on the sample level using positive and negative control populations was applied. However, the batch effect persisted for CD69 expression by ASCs, resulting in the exclusion of CD69 from further analysis. ASC maturity is systematically underestimated by the ASC-ME model when comparing values of predicted and actual DPI. This underestimation likely originate from overrepresentation of early immune stages in the training and validation datasets, as many included studies focused on early immune responses. Alternatively, this underestimation (but also the variance in predicted ASC maturity mentioned previously) could also have resulted from other unrelated but overlapping immune reactions during the sampling periods such as unreported infections or vaccinations. This hypothesis is supported by the observation that most of the included datasets involve influenza vaccination, typically administered in autumn, a season associated with increased infection risk. Furthermore, ASC maturation likely follows a saturation curve with plateau, suggesting completion of ASC maturation, at least in the blood, at later immune stages approximately 150 days after response initiation. As additional general limitations, our study was not able to gather enough data to evaluate additional maturity markers but could only focus on a limited number of eight markers, all previously implied to be relevant in ASC maturation. Since our study is based solely on publicly available data, functional validation of our ASC-ME model requires further investigation. The ideal experimental design for our model would be a timestamped vaccination without natural occurring recall, like smallpox vaccination, combined with a standardized readout of vaccine-specific ASCs for their expression patterns in blood and bone marrow at various time points post vaccination.

## Resource availability

### Lead contact

Requests for further information and resources should be directed to and will be fulfilled by the lead contact, Tobit D. Steinmetz (d.t.steinmetz@umcg.nl).

### Materials availability

This study did not generate new unique reagents.

### Data and code availability

• Flow cytometry data have been deposited at http://flowrepository.org as FR-FCM-Z8QH (http://flowrepository.org/experiments/9041) and FR-FCM-Z8QW (http://flowrepository.org/experiments/9042), respectively, and are publicly available as of the date of publication. This paper analyzes existing, publicly available data, accessible at http://flowrepository.org/, https://immport.org/ or https://cytobank.org/. An overview of all publicly available datasets considered or used in this study is given in Table S2.
• All original code has been deposited at https://github.com/rki-umcg/ASC_ME and is publicly available as of the date of publication. In order to use the ASC-ME model, an online application (accessible via https://steinmetz.shinyapps.io/asc_me/) was built guiding the user through all necessary steps to use the ASC-ME model with own data. A detailed annotated code is available in this paper’s supplemental information (see Document S1).
• Any additional information required to reanalyze the data reported in this paper is available from the lead contact upon request.

## Acknowledgments

Several authors of this publication are members of the European Reference Network ReCONNET. We thank all researchers who openly share their data via unrestricted online repositories as prerequisite for this type of study, especially Ryan Baxter and Elena Hsieh for sharing their SLE patient data. This work is funded by a grant from the 10.13039/501100001659German Research Foundation (10.13039/501100001659Deutsche Forschungsgemeinschaft, DFG)—project no. 456067883 to T.D.S. This publication is part of project numbers 09150162010166 and 09150162210226 of the Veni research program to G.M.V. and T.D.S., respectively, which are financed by the 10.13039/501100003246Dutch Research Council (10.13039/501100003246NWO). Additional funding for this project was received from the Junior Talent Call Program from the 10.13039/100018286Dutch Arthritis Society (ReumaNederland) with grant no. JTC23-1-401 to T.D.S. All funding sources were unrestricted and had no role in study design, collection, analysis or interpretation of study data or writing.

## Author contributions

T.D.S., G.M.V., and F.G.M.K. jointly designed the project. T.D.S., G.Y., T.d.N., N.v.d.M., and N.K. performed the experiments. T.D.S., G.M.V., H.B., and F.G.M.K. supervised the work. T.D.S. wrote the initial manuscript. T.D.S., R.W., and G.Y. analyzed the data. All authors have read and critically revised the manuscript.

## Declaration of interests

The authors declare no competing interests.

## Declaration of generative AI and AI-assisted technologies in the writing process

During the preparation of this work the authors used ChatGPT (version 4, v.2) in order to partially draft or troubleshoot R scripts. After using this tool, the authors reviewed and edited the content as needed and take full responsibility for the content of the publication.

## STAR★Methods

### Key resources table

**Table:** 

REAGENT or RESOURCE	SOURCE	IDENTIFIER
**Deposited data**		

Cytometry datasets	flowrepository	http://flowrepository.org/
Cytometry datasets	Immport	https://immport.org/
Cytometry datasets	Cytobank	https://cytobank.org/
plasma cell characterization in Sjögrens disease patients and healthy individuals following SARS-CoV2 vaccination	This paper; flowrepository	http://flowrepository.org/experiments/9041
plasma cell characterization in patients with systemic rheumatic diseases	This paper; flowrepository	http://flowrepository.org/experiments/9042
See Table S2 for an overview of all deposited data used.		

**Software and algorithms**		

ASC-ME model	This paper	https://github.com/rki-umcg/ASC_ME
RStudio 2023.06.1 Build 524 or 2025.05.1 Build 513	Posit Software	https://dailies.rstudio.com/version/2023.06.1+524/ https://dailies.rstudio.com/version/2025.05.1+513/
InkScape 1.4.3	InkScape Developers	www.inkscape.org

### Experimental model and study participant details

The datasets analyzed in this study consist of various compositions regarding age, sex and ethnicity. An overview of the participant details is provided in Table S2.

### Method details

The development and evaluation of the ASC-ME prediction model adheres to the recommendations outlined by the TRIPOD+AI guidelines^35^ for prediction models using machine learning and the TRIPOD-Cluster guidelines^36^ for studies involving multiple datasets.

#### Repository search and selection

Three data repositories, namely http://flowrepository.org/, https://immport.org/ and https://cytobank.org/, were screened for eligible datasets between March 2023 and July 2024 using keywords adjusted to the respective repository, including: “bone marrow”, “PBMC”, “gut”, “spleen”, “lymph node”, “tonsil”, “lung”, “kidney”, “plasma cell”, “B cell”, “vaccine”, “infection”, “lupus”, “sjögren”, “sclerosis”, “arthritis”, “myositis”, “psoriasis”, “colitis”, “myasthenia gravis”, “diabetes”, “CyTOF”, “flow cytometry”, and “mass cytometry” for *flowrepository* and *immport* and all data was screened at *Cytobank* (171 datasets). To be included in this study datasets must contain human samples that are available for download and are stained for expression of CD3, CD27, CD38, CD19 with at least one additional potential antibody-secreting cell maturity marker (Table S1). In the second step, datasets were downloaded and evaluated to ensure that sample files and annotation data could be aligned and that the sample files contained all relevant cell populations (T-cells, B-cells, ASCs, respective control populations). Additional datasets locally generated at the University Medical Center Groningen (UMCG) that fulfilled the criteria above were added. Samples with a subject age lower than ten years were excluded, due to frequently occurring infections and the ongoing development of the immune system in children. Also samples with an ASC count lower than 50 cells were excluded to increase reliability of the MFI-based expression readout. A total of 4257 samples within 39 datasets were included in this study (overview in Table S2).

#### Processing and standardization of cytometry samples

Downloaded datasets were analyzed using a RStudio code pipeline individually adjusted to the respective dataset (see Supplemental Appendix for a detailed description and section *Code availability*). Major adjustments between datasets concerned usage of DNA double-staining for mass cytometry or usage of forward and sideward scatter for flow cytometry as the initial sample cleanup step (Figure S1A), alignment of cell marker with the used fluorophores or metals, detailed positions of gates and gating of reference populations based on the maturity marker present in a respective sample.

The full procedure to process an example dataset is given in the Supplemental Appendix. In brief, processing, gating, standardization and extraction of potential antibody-secreting cell maturity markers from the datasets included the following steps: 1) import fcs-files into RStudio and evaluation of the sample compensation. Recompensation was required in 9 out of 38 datasets and performed using compensation matrices stored within the fcs-files themselves; (2) rename and standardize detection channels according to the used cellular markers in the respective dataset; (3) gating of the main cell populations as clean, single and viable cells followed by T-cells, B-cells and ASCs (Figure S1A); (4) define relevant control populations depending on the respective marker (Table S1); (5) extract median fluorescence intensities (MedFI) for each sample and population; (6) calculate relative expression for each potential maturity marker using the respective control population (Figure 2A; Figure S1B); and (7) generate overlay plots of control cells and ASCs for each potential maturity marker as output quality control and export all data for the respective dataset.

Output tables from all datasets were combined and annotated further, e.g., for sample age and gender by merging cytometry output and metadata. Samples with less than 50 identified ASCs or a subject age younger than ten years were removed. The resulting data was filtered for studies with controlled and timed immune kinetics up to a follow-up time of 180 days post vaccination or infection. All available kinetic data (1941 samples from 23 datasets) were split, on a dataset basis, into a training subset (75% of data) and a validation subset (25% of data). Thereby, mean study follow-up time was the main parameter to balance between training and validation subsets with 13.93±28.55 and 14.34±28.55 days of follow-up respectively. Age, sex and race were secondary parameters to balance the split (Figure S2).

#### Setup for the maturity prediction model using random forest

To set up a prediction model, random forest-based machine learning (see the Supplemental Appendix in Document S1 for the detailed approach with annotated code) was chosen, since random forest models are suitable for continuous outcomes, resistant to overfitting and cope well with non-linear input variables. A total sum of 1064 samples from 23 datasets (Figure 1) from controlled infection and vaccination of healthy human individuals contained annotations of the day post immune-intervention (DPI) ranging from five to 180 days (i.e., kinetic samples). A total of 17 datasets (795 samples) were used for training the model and six datasets (269 samples) for external validation. Based on the training set, a random forest prediction model was trained on predicting the DPI-timepoint of a sample based on various potential antibody-secreting cell maturity markers, namely CD19, CD20, CD28, CD45, CD56, CD138, HLA-DR, and Ki67 as final predictor setup. In brief, the following steps were performed: (1) trim the training data to omit excluded maturity markers and normalized expression values outside the range of −2 to 5; (2) missing data (28.7% of total data) on maturity markers were imputed using the *mice* package with the random forest method for 20 iterations yielding a bundle of 20 imputed output tables; (3) a random forest prediction model was trained using the imputed data bundle (15,900 data entries) to predict the DPI of a sample based on predictors mentioned in Table S1; (4) several models using various predictor combinations were trained and evaluated based on the R value of a Pearson correlation with actual DPI and predicted DPI. Four predictors (CD44, BCL-2, BCMA, and TACI) were directly omitted due to their high percentage of missing data and absence in the validation dataset. Age, sex, CD69, and CD86 were tested in various alterations of the prediction model but lacked added value. Additionally, values for parameters like ntree = 100|200|300|500 and mtry = 3|4|5|6|7 were tested and evaluated within the model development. However, the number of trees had no effect on the model performance (data not shown); (5) a final random forest model using eight predictors mentioned above, with the final setting of ntree = 100 and mtry = 6 was chosen.

### Quantification and statistical analysis

*The Mann-Whitney U-test was used for statistical analysis with two groups, while three or more groups were analyzed with a one-way ANOVA with Tukey correction or with a Kruskal-Wallis test with Bonferroni correction. Correlations were tested using Pearson correlation.* Terminology for correlation coefficients is used according to Chan et al.^37^: *poor* for r < 0·3, *fair* for 0·3 < r < 0·5, *moderate* for 0·6 < r < 0·8, and *very strong* for r > 0·8.

### Additional resources

ASC-ME is an open accessible, ready-to-use online tool (https://steinmetz.shinyapps.io/asc_me/). A guide and data template are provided to assist users in adapting and analyzing their own data with the ASC-ME model including automated generation of plots and download of processed user data for further analysis.

## Supplemental information

Document S1. Figures S1–S10, Table S1, and Methods S1

Table S2. Detailed overview of all studies used for development, validation, and application of the ASC-ME model, related to Figure 1 and STAR Methods
