# Supplementary material for: Development, validation, and implementation of the antibody-secreting cell maturity index: Universal prediction of human plasma cell maturity
Source: iScience. 2026 May 22;29(6):116050. doi: 10.1016/j.isci.2026.116050 (PMC13217880; doi:10.1016/j.isci.2026.116050)
Supplement: Document S1. Figures S1–S10, Table S1, and Methods S1 [file mmc1.pdf]

**iScience, Volume *29***

## Supplemental information

# Development, validation, and implementation of the antibody-secreting cell maturity index: Universal prediction of human plasma cell maturity

**Tobit D. Steinmetz, Guiyou Yang, Tessa de Nooijer, Nisha van der Meer, Naomi Karmi, Rick Wilbrink, Hendrika Bootsma, Gwenny M. Verstappen, and Frans G.M. Kroese**

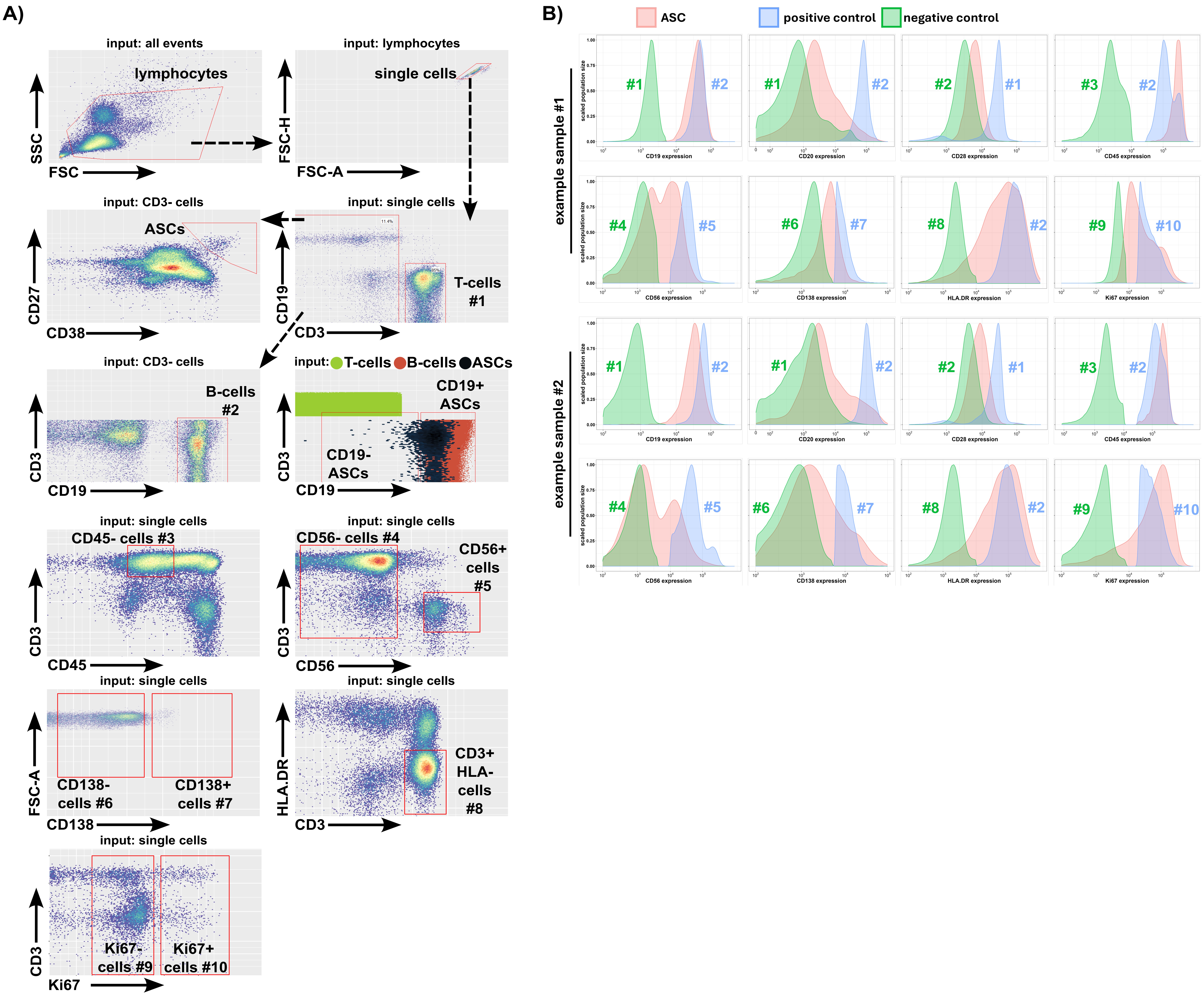


***Supplementary Figure S1:*** *Processing of cytometry data.* ***A)*** *Consecutive gating of lymphocytes, single cells T- and none-T-cells, B-cells, and antibody-secreting cells (ASC), a T-cell, B-cell and ASCs overlay gating CD19+ and CD19- ASCs in an exemplary sample and additional control populations (numbered using #).* ***B)*** *overlay histograms for expression of all maturity markers by ASCs and control populations in two exemplary samples. Related to Figure 1 and 2.*

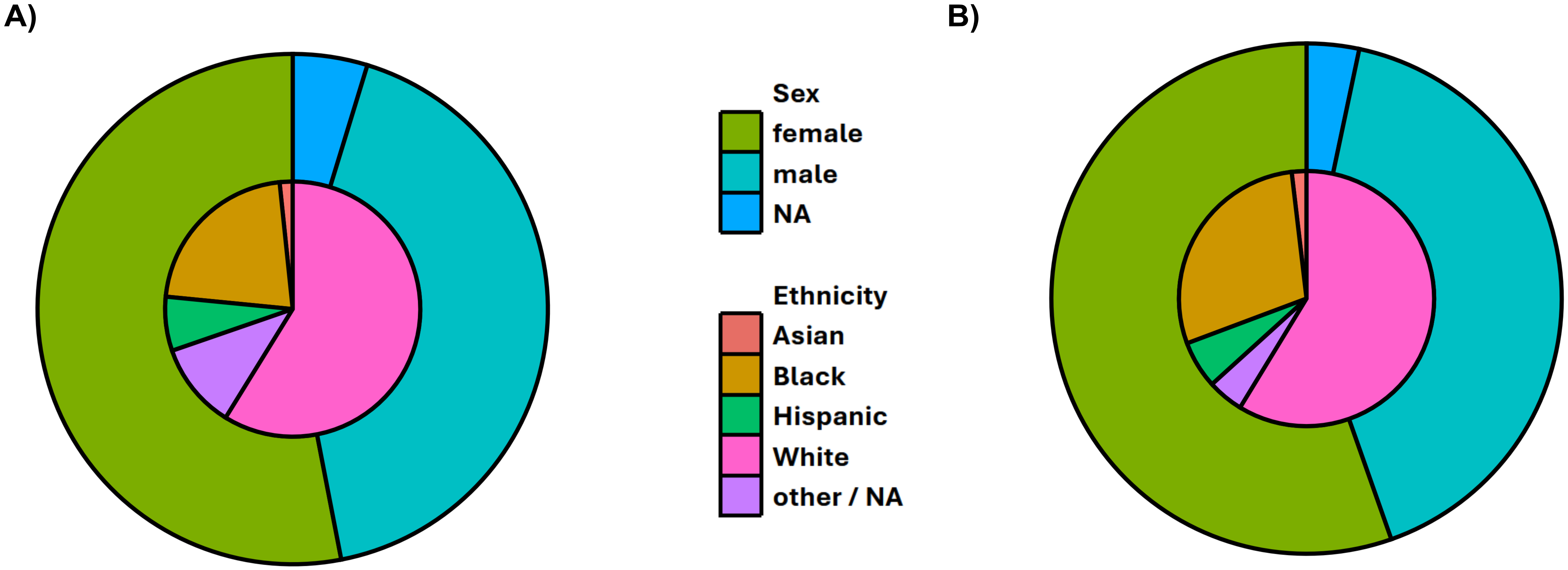


***Supplementary Figure S2:*** *Overview of ethnicity (inner circles) and sex (outer circles) distribution within the used* ***A)*** *training datasets and* ***B)*** *validation datasets. Related to Figure 1.*

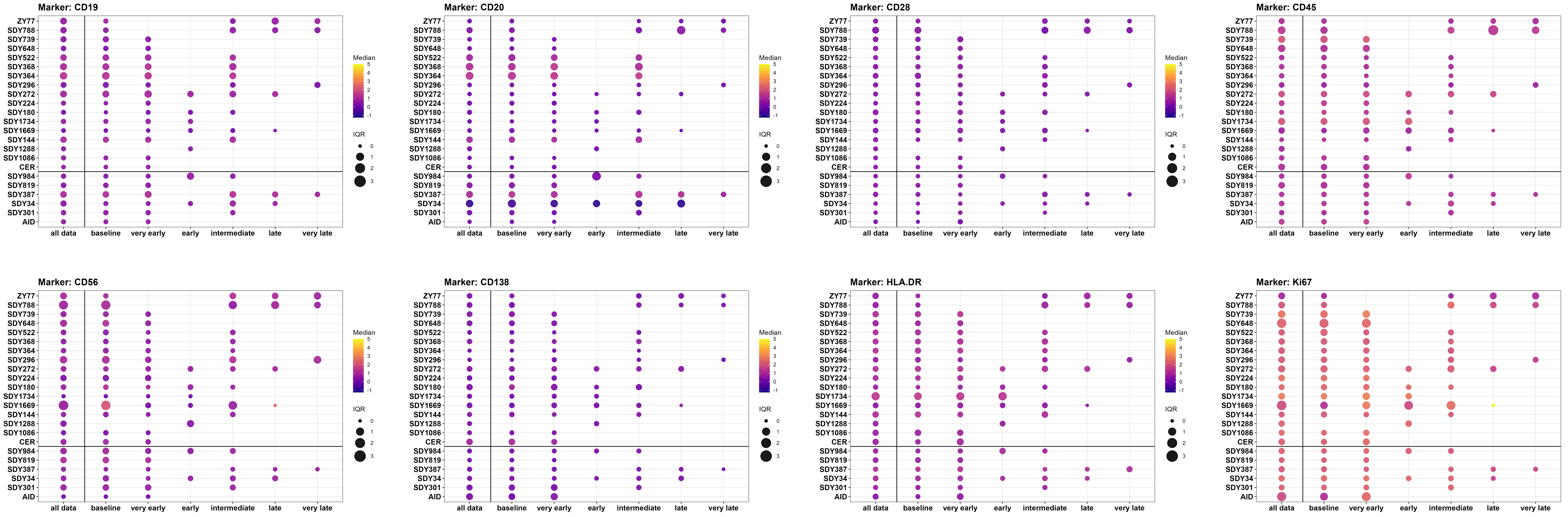


***Supplementary Figure S3:*** *Overview of marker homogeneity across all individual studies used for training (upper 17 studies of each plot) and validation (lower 6 studies of each plot), for all markers and longitudinal immune stages, represented as dot plots with median (dot color) and interquartile range (IQR, dot size). Related to Figure 2.*

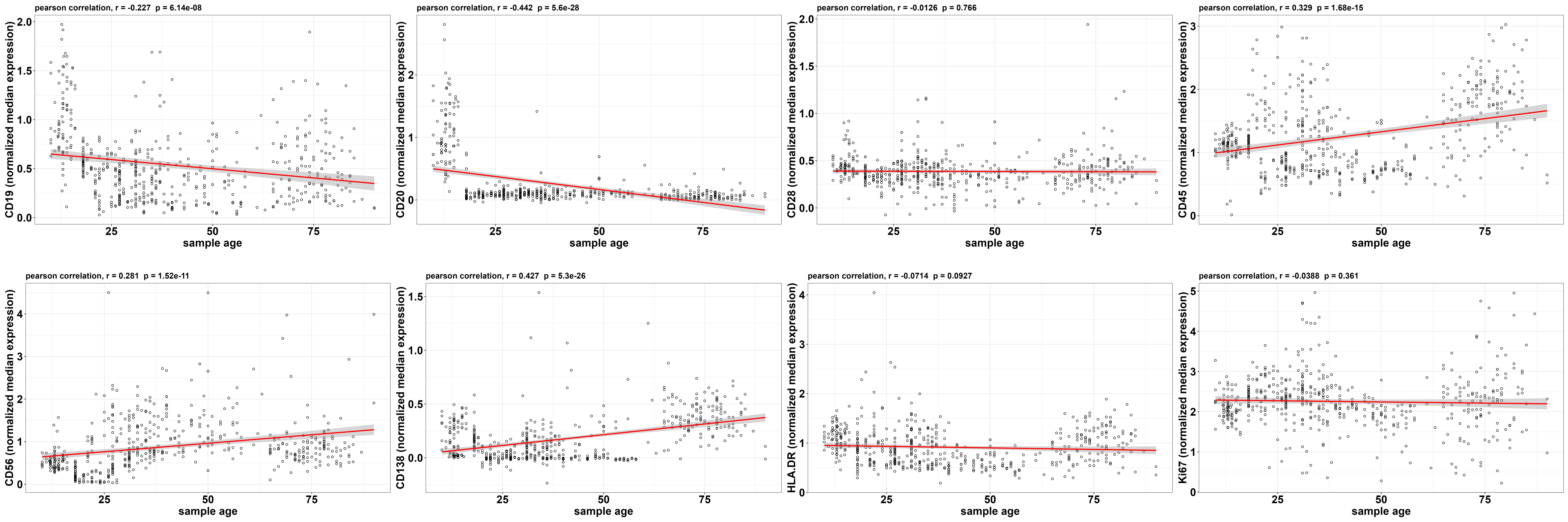


***Supplementary Figure S4:*** *Normalized median expression of CD19, CD20, CD28, CD45, CD56, CD138, HLA-DR and Ki67 by total blood antibody-secreting cells in 664 baseline samples in relation to the individuals age at the sampling timepoint. P-and r-values calculated using Pearson correlation with linear regression and 95% confidence intervals (red line with grey area). Related to Figure 2.*

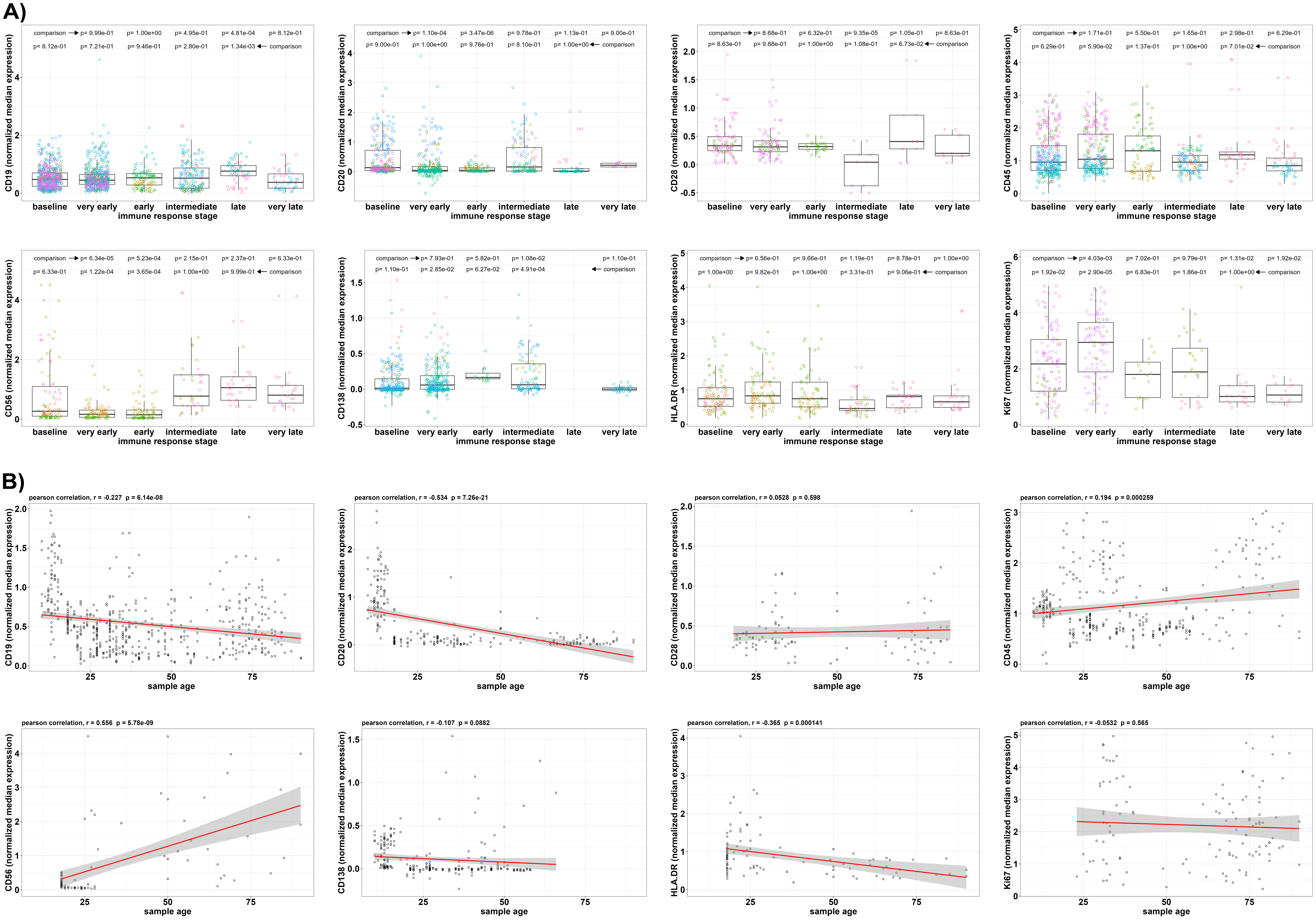


***Supplementary Figure S5:*** *Normalized median expression of maturity markers by total blood antibody-secreting cells* ***A)*** *during a timed immune reaction in unimputed longitudinal samples and* ***B)*** *in unimputed baseline samples in relation to the individuals age at the sampling timepoint. Data point coloring according to individual datasets and box plots represented as median with 95% confidence intervals. P-values calculated using one-way ANOVA with Tukey correction in A) and using Pearson correlation in B) with linear regression and 95% confidence intervals (red line with grey area). Related to Figure 2.*

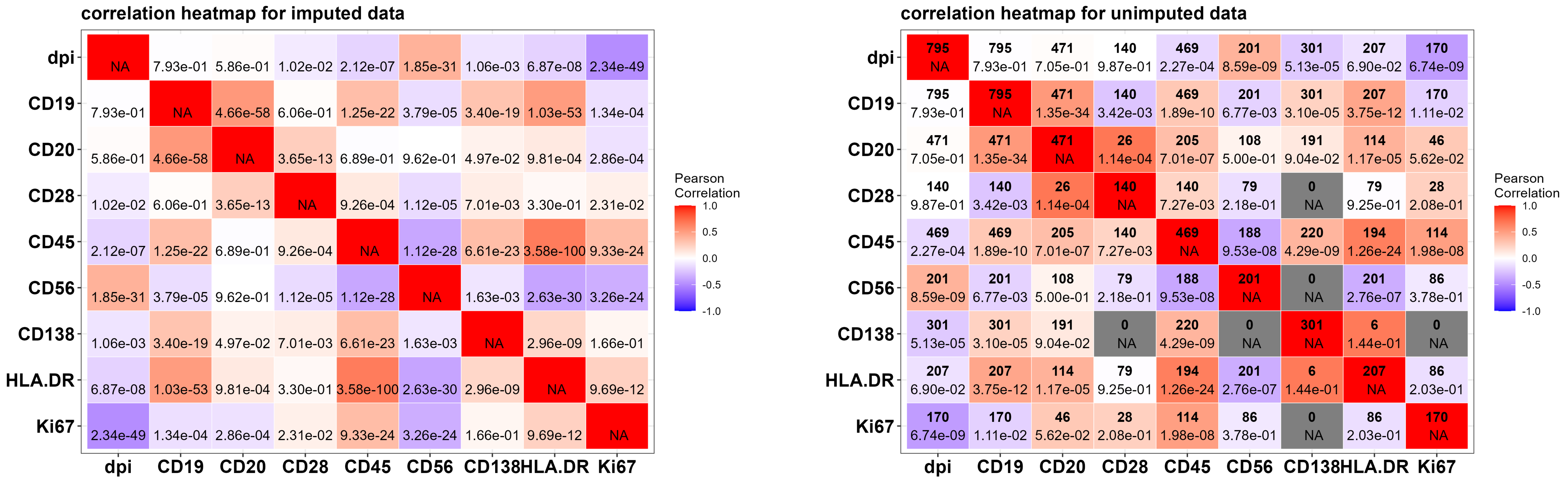


***Supplementary Figure S6:*** *Correlation matrices for DPI and maturity markers prior to data imputation (right panel) and with data imputation (left panel). Color gradient according to the strength of the Pearson correlation r-value. Upper number in boxes indicate sample numbers for the respective correlation (n=795 for all imputed correlation pairs) and lower number display the correlation p-value. Related to Figure 3.*

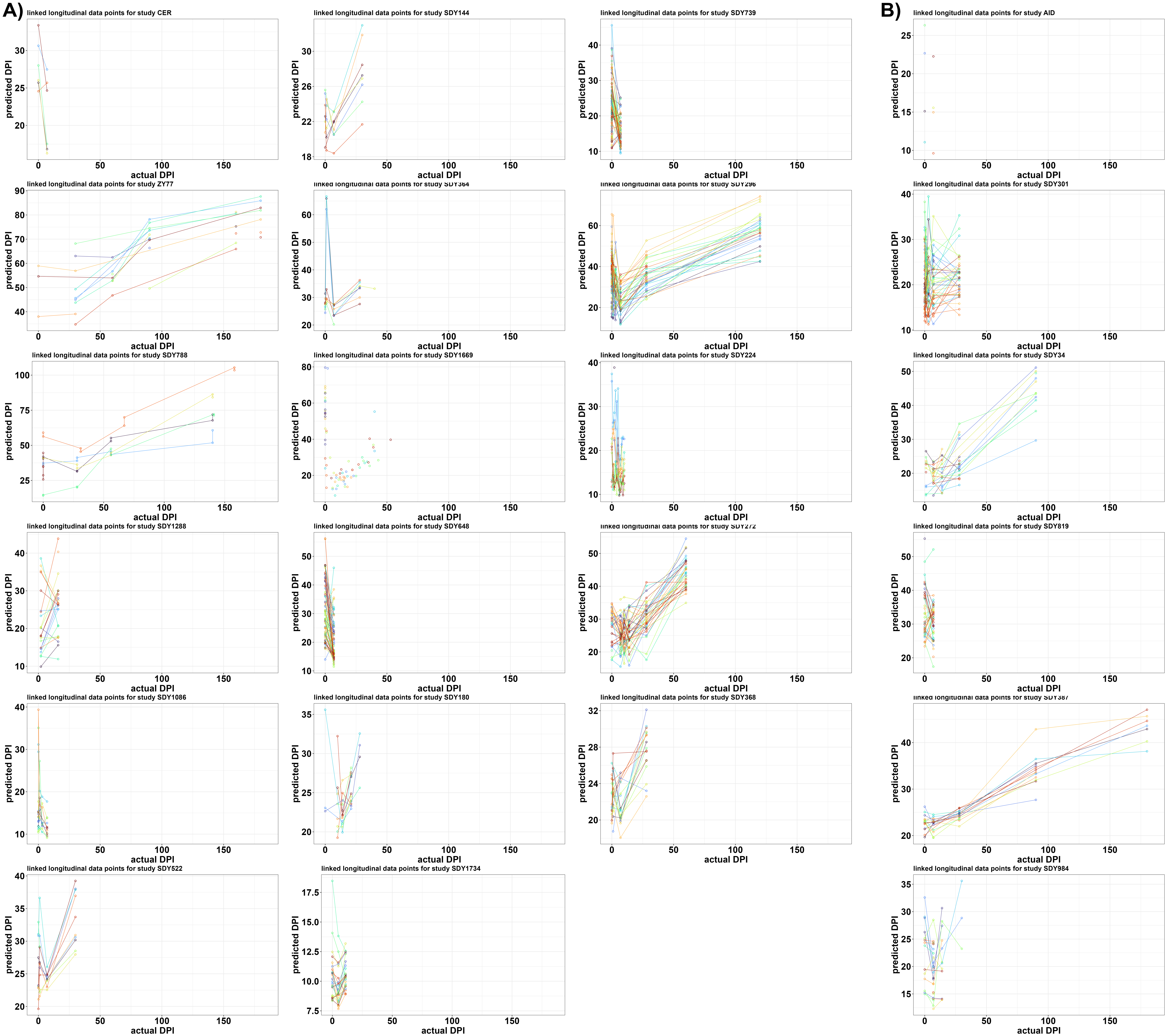


***Supplementary Figure S7:*** *Longitudinal data of actual DPI and predicted DPI during timed immune reactions for each study separately involved in A) model training and B) model validation; data points from the same individuals are connected. Related to Figure 3.*

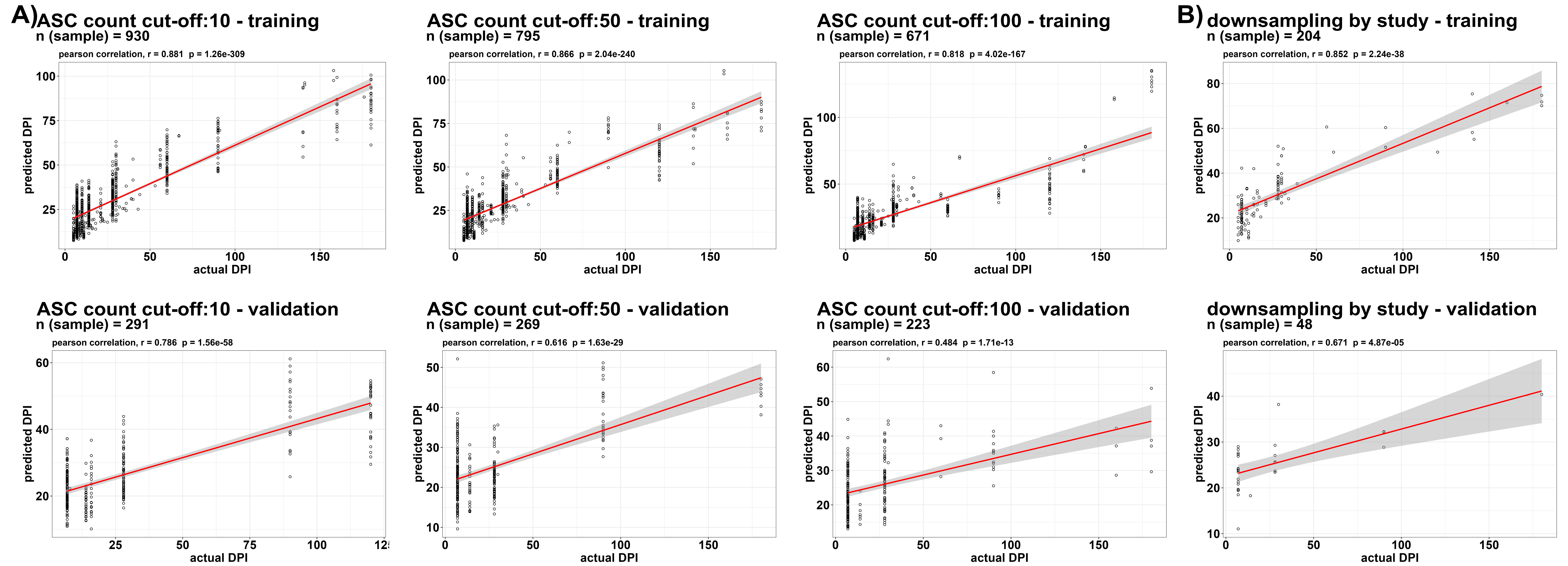


***Supplementary Figure S8: A)*** *Comparison of model training (upper panel) and model validation (lower panel) using different cut-offs values for ASC counts, namely, ASC count ≥10 (left panel), ASC count ≥50 (middle panel) and ASC count ≥100 (right panel).* ***B)*** *Model training and validation using down-sampled data with equal datapoints per study. P-and r-values calculated using Pearson correlation with linear regression and 95% confidence intervals (red line with grey area). Related to Figure 3.*

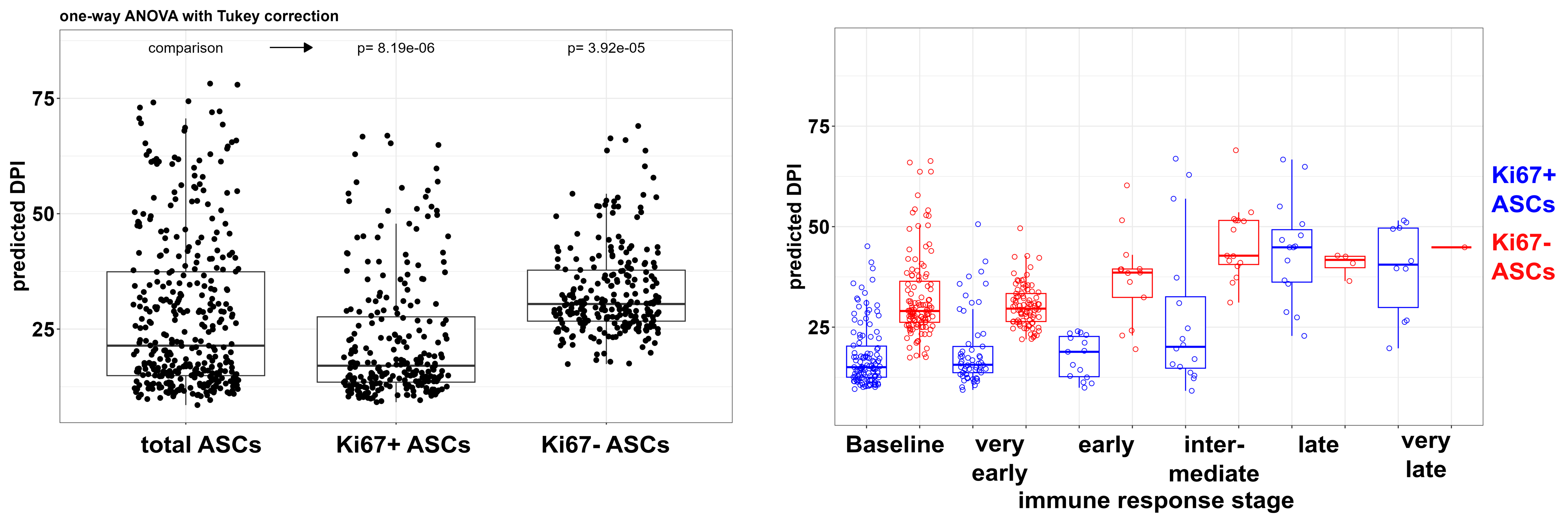


group comparison

| | | |
|---|---|---|
| Ki67+ baseline | Ki67- baseline | p= 1.47e-21 |
| Ki67+ very early | Ki67- very early | p= 1.86e-09 |
| Ki67+ early | Ki67- early | p= 1.42e-04 |
| Ki67+ intermediate | Ki67- intermediate | p= 3.05e-04 |
| Ki67+ late | Ki67- late | p= 1.00 |
| Ki67+ very late | Ki67- very late | p= 1.00 |
| Ki67+ baseline | Ki67+ very early | p= 1.00 |
| Ki67+ baseline | Ki67+ early | p= 1.00 |
| Ki67+ baseline | Ki67+ intermediate | p= 0.798 |
| Ki67+ baseline | Ki67+ late | p= 2.22e-11 |
| Ki67+ baseline | Ki67+ very late | p= 3.52e-06 |
| Ki67- baseline | Ki67- very early | p= 1.00 |
| Ki67- baseline | Ki67- early | p= 1.00 |
| Ki67- baseline | Ki67- intermediate | p= 0.031 |
| Ki67- baseline | Ki67- late | p= 1.00 |
| Ki67- baseline | Ki67- very late | p= 1.00 |

***Supplementary Figure S9:*** *Assessment of Ki67- and Ki67+ ASC subpopulations at all timepoints in comparison to total ASCs, represented as box plots with median and 95% confidence intervals using one-way ANOVA with Tukey correction (left panel). Ki67- and Ki67+ ASC subpopulations during ongoing immune reactions (middle panel), represented as box plots with median and 95% confidence intervals compared with a Kruskal-Wallis test with Bonferroni correction (right panel). Related to Figure 5.*

## An analogue model without Ki67 as prediction marker:

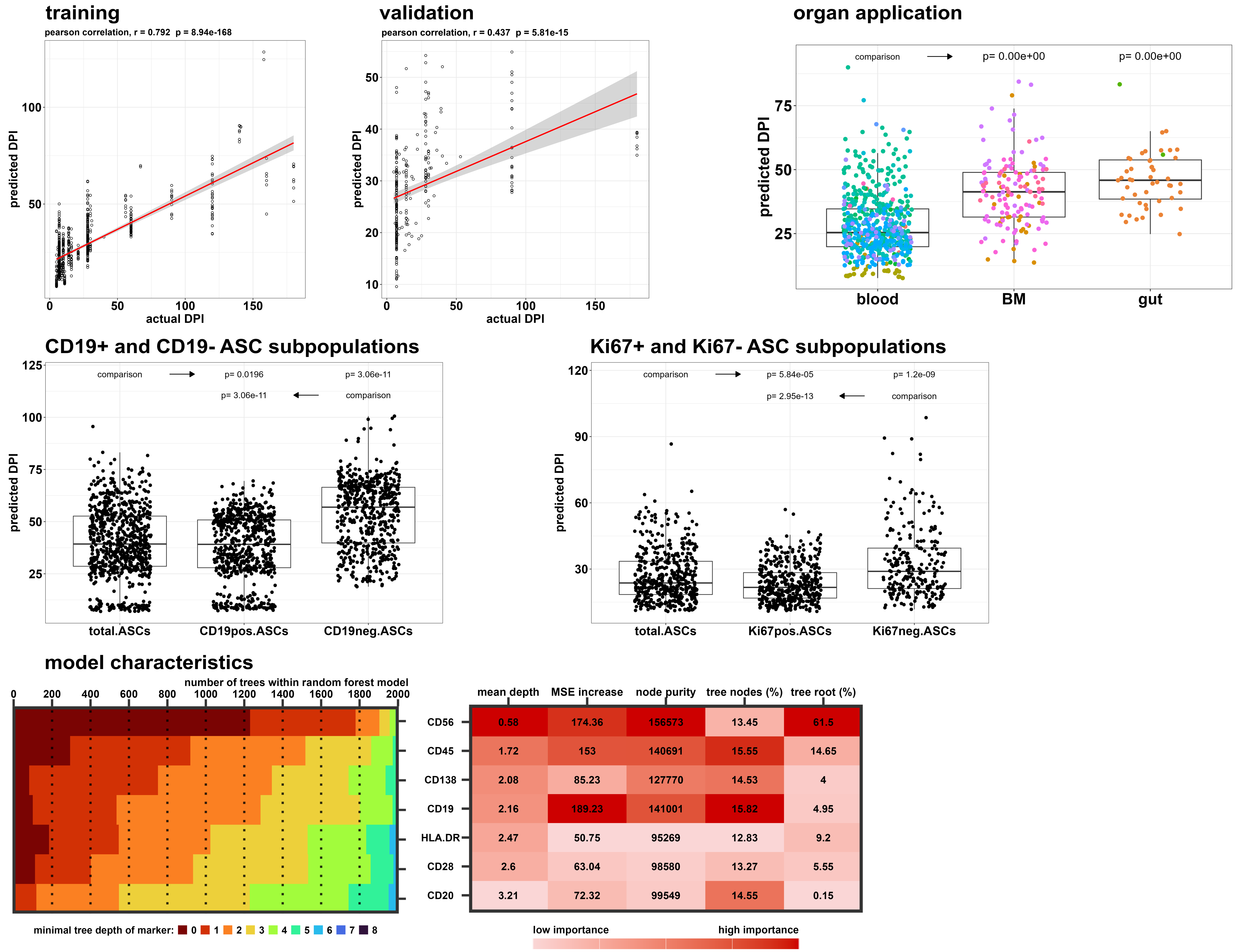


***Supplementary Figure S10:*** *Overview of an alternative maturity prediction model without Ki67 as prediction marker during model training, validation, application to organ samples, CD19 and Ki67 based subpopulations and model characteristics. Related to Figure 5. For scatter plots, p-and r-values calculated using Pearson correlation with linear regression and 95% confidence intervals (red line with grey area). For box plots, data point coloring according to individual datasets and represented as median with 95% confidence intervals. P-values calculated using one-way ANOVA with Tukey correction.*

**Table S1. Overview of potential antibody-secreting cell maturity markers and reference populations used for internal normalization of datasets. Gray background = maturity markers selected as predictors for the final ASC-ME model. Related to Figure 2.**

| potential maturity marker | Positive Control | Negative Control | completeness training set (n = 1459) before outlier removal n | % | after outlier removal n | % | completeness validation set (n = 482) before outlier removal n | % | after outlier removal n | % | usage in RF model | remark |
|---|---|---|---|---|---|---|---|---|---|---|---|---|
| dpi | n/a | n/a | 1459 | 100,0 | 1459 | 100,0 | 482 | 100,0 | 482 | 100,0 | - | outcome value |
| CD19 | CD19+ B-cells | CD3+ T-cells | 1459 | 100,0 | 1459 | 100,0 | 482 | 100,0 | 482 | 100,0 | yes | suspected negative maturity marker |
| CD20 | CD19+ B-cells | CD3+ T-cells | 803 | 55,0 | 799 | 54,8 | 269 | 55,8 | 262 | 54,4 | yes | suspected negative maturity marker |
| CD28 | CD3+ T-cells | CD19+ B-cells | 242 | 16,6 | 242 | 16,6 | 8 | 1,7 | 8 | 1,7 | yes | suspected positive maturity marker |
| CD44 | live+CD44+ cells | live+CD44- cells | 89 | 6,1 | n/a | n/a | 0 | 0,0 | n/a | n/a | no | omitted: only in one datset; number of missing values |
| CD45 | CD19+ B-cells | live+CD14+ cells | 888 | 60,9 | 888 | 60,9 | 280 | 58,1 | 280 | 58,1 | yes | suspected negative maturity marker |
| CD56 | CD16+CD56+ cells | live+CD56- cells | 332 | 22,8 | 328 | 22,5 | 8 | 1,7 | 8 | 1,7 | yes | suspected positive maturity marker |
| CD69 | CD14+CD69+ cells | live+CD69- cells | 218 | 14,9 | n/a | n/a | 26 | 0,1 | n/a | n/a | tested, omitted | omitted: unclear batch effect |
| CD86 | CD19+ B-cells | live+CD86- cells | 1136 | 77,9 | n/a | n/a | 77 | 0,2 | n/a | n/a | tested, omitted | omitted: no added predictive value |
| CD138 | live+CD138+ cells | live+CD138- cells | 631 | 43,2 | 631 | 43,2 | 357 | 74,1 | 357 | 74,1 | yes | suspected positive maturity marker |
| HLA.DR | CD19+ B-cells | CD3+HLA- cells | 344 | 23,6 | 344 | 23,6 | 8 | 1,7 | 8 | 1,7 | yes | suspected negative maturity marker |
| Ki.67 | live+Ki67+ cells | live+Ki67- cells | 362 | 24,8 | 291 | 19,9 | 8 | 1,7 | 8 | 1,7 | yes | suspected negative maturity marker |
| Bcl-2 | CD3+ T-cells | live+Bcl2- cells | 0 | 0,0 | n/a | n/a | 0 | 0,0 | n/a | n/a | no | omitted: not available in blood samples |
| BCMA | CD19+ B-cells | live+BCMA- cells | 0 | 0,0 | n/a | n/a | 0 | 0,0 | n/a | n/a | no | omitted: not available in blood samples |
| TACI | CD19+ B-cells | live+TACI- cells | 0 | 0,0 | n/a | n/a | 0 | 0,0 | n/a | n/a | no | omitted: not available in blood samples |
| **other marker** | | | | | | | | | | | | |
| Age | n/a | n/a | 1459 | 100,0 | 1459 | 100,0 | 482 | 100,0 | 482 | 100,0 | tested, omitted | omitted: no added predictive value |
| Sex | n/a | n/a | 1390 | 95,3 | 1390 | 95,3 | 466 | 96,7 | 466 | 96,7 | tested, omitted | omitted: no added predictive value |

# Method S1 – In-detail annotated code for cytometry data analysis, development and validation of the ASC-ME model

### *Processing and standardization of cytometry samples*

Datasets were evaluated to ensure that sample files were sufficiently annotated with metadata and that the samples files contained all relevant cell populations (T-cells, B-cells, ASCs, respective control populations). For flow cytometry samples, various fluorophore combinations (like FITC and PE) were evaluated to inspect whether the sample data requires compensation. In case insufficient compensation was suspected, the fcs-file internal compensation matrix was read-out and applied to the whole dataset before continuing.

Cytometry data was processed and analyzed as follows (using a flow cytometry dataset as example):

```
# code to analyze flow and mass cytometry data to generate normalized expression values for human ASCs
# load the libraries required for this project step
lapply(list('flowCore','flowAI','ggplot2','ggcyto','flowViz','openCyto','flowWorkspace','gridExtra','dplyr','writexl'), require, character.only = TRUE)

# specify location of fcs-files to analyze and load them into a flow-set (fs) and gating-set (gs)
files <- list.files(path="…/ASC_ME_project/CER", pattern = ".FCS", ignore.case = TRUE)
fs_all <- read.flowSet(files, path="…/ASC_ME_project/CER", truncate_max_range = FALSE)
gs_all<-GatingSet(fs_all)
# set an output path for all related export files
output_path="…/ASC_ME_output/CER"

# use autoplot to control the presence of all expected cytometry marker/parameter
autoplot(fs_all[[1]])

# evaluate if compensation data is present within the fcs-file meta-data. E.g., using:

ggcyto(gs_all[[1]],aes(x="FITC-A",y="PE-A"))+geom_hex(bins = 512)+gggcyto_par_set(limits = "instrument")+scale_x_logicle()+scale_y_logicle()
# apply the compensation matrix to the gating-set if necessary (otherwise skip the following three steps):

comp_matrix<-keyword(gs_all[[1]])$SPILL
comp<-compensation(comp_matrix)
gs_all<-compensate(gs_all, comp)
```

Rename and standardize channels: names of channels need to be adapted to every dataset individually (based on the readout using *autoplot*).

```
# rename gating marker to adjust marker to respective channel/metal
# select/un-select required markers using '#'
colnames(gs_all)[colnames(gs_all)=="FITC-A"] <- "CD3"
#colnames(gs_all)[colnames(gs_all)=="Ce142Di"] <- "CD14"
#colnames(gs_all)[colnames(gs_all)=="R730/45-710-A"] <- "CD16"
colnames(gs_all)[colnames(gs_all)=="BV650-A"] <- "CD27"
colnames(gs_all)[colnames(gs_all)=="APC-eFluor 780-A"] <- "CD38"
#colnames(gs_all)[colnames(gs_all)=="Ir191Di"] <- "DNA1"
```

```r
#colnames(fs_all)[colnames(fs_all)=="Ir191Di"] <- "DNA1"
#colnames(gs_all)[colnames(gs_all)=="Ir193Di"] <- "DNA2"
#colnames(fs_all)[colnames(fs_all)=="Ir193Di"] <- "DNA2"
colnames(gs_all)[colnames(gs_all)=="BV510-A"] <- "viability"
#colnames(gs_all)[colnames(gs_all)=="Ce140Di"] <- "Beads"

# rename maturity marker
colnames(gs_all)[colnames(gs_all)=="BUV737-A"] <- "CD19"
colnames(gs_all)[colnames(gs_all)=="Alexa Fluor 700-A"] <- "CD20"
#colnames(gs_all)[colnames(gs_all)=="Gd158Di"] <- "CD28"
colnames(gs_all)[colnames(gs_all)=="BUV563-A"] <- "CD45"
#colnames(gs_all)[colnames(gs_all)=="V710/50-680-A"] <- "CD56"
colnames(gs_all)[colnames(gs_all)=="PE-Cy5-A"] <- "CD138"
colnames(gs_all)[colnames(gs_all)=="PE-Fire810-A"] <- "HLA.DR"
#colnames(gs_all)[colnames(gs_all)=="Er168Di"] <- "Ki67"

# control successful application of new channel names
all.set.marker<-c("CD19","CD20","CD45","CD138", "HLA")
colnames(gs_all)
```

Proceed with gating the required cell populations. The general steps include defining the borders of the gate, check and adapt the gate using multiple scatter plots, generate and save an overview plot for all files for quality control. Lastly, apply the gate to the gating-set and recompute the changes.

```r
# 1. cleaning gate (FSC/SSC for flow and DNA1/DNA2 for mass cytometry)
g.clean<-polygonGate("FSC-A"=c(5e5,1e6,2e6,2e6,5e5,4e5),"SSC-A"=c(0,0,5e5,2.5e6,2e6,1e6), filterId = "g.clean") #set gate coordinates & name gates
ggcyto(fs_all[[1]],aes(x="FSC-A",y="SSC-A"))+geom_hex(bins = 256)+geom_gate(g.clean)+ggcyto_par_set(limits = "instrument") #evaluate gate in one sample

ggcyto(fs_all[[3]],aes(x="FSC-A",y="SSC-A"))+geom_hex(bins = 256)+geom_gate(g.clean)+ggcyto_par_set(limits = "instrument") #evaluate gate in another sample

ggcyto(fs_all,aes(x="FSC-A",y="SSC-A"))+geom_hex(bins = 256)+geom_gate(g.clean)+ggcyto_par_set(limits = "instrument") #plot all samples with respective gate

ggsave("lymph_plot.png",height = 4000, width = 6000,units = "px", path = output_path) #save QC overview plot for all samples
gs_pop_add(gs_all, g.clean, parent="root") #add to gating set
recompute(gs_all) #apply changes

# 2.live gate
g.live<-polygonGate("FSC-A"=c(4e6,4e6,2e5,2e5),"viability"=c(2e4,900,900,2e4), filterId = "g.live")
ggcyto(gs_all[[1]],aes(x="FSC-A",y="viability"), subset = "g.clean")+geom_hex(bins = 512)+geom_gate(g.live)+scale_x_logicle()+scale_y_logicle()+ggcyto_par_set(limits = "instrument") #check gate

ggcyto(gs_all[[3]],aes(x="FSC-A",y="viability"), subset = "g.clean")+geom_hex(bins = 512)+geom_gate(g.live)+geom_stats(adjust = 1)+scale_x_logicle()+scale_y_logicle()+ggcyto_par_set(limits = "instrument") #check gate

ggcyto(gs_all,aes(x="FSC-A",y="viability"), subset = "g.clean")+geom_hex(bins = 512)+geom_gate(g.live)+geom_stats(adjust = 1)+scale_x_logicle()+scale_y_logicle()+ggcyto_par_set(limits = "instrument")
```

```
ggsave("via_length_plot.png",height = 4000, width = 6000,units = "px", path = output_path)
gs_pop_add(gs_all, g.live, parent="g.clean")
recompute(gs_all)

# 3. T-cell and none T-cell gate
# T-cell gate
g.Tcells<-polygonGate("CD3"=c(7000,1e5,1e5,7000),"CD19"=c(-400,-400,900,900), filterId = "g.Tcells")
ggcyto(gs_all[[1]],aes(x="CD3",y="CD19"), subset = "g.live")+geom_hex(bins = 512)+geom_stats(adjust = 1)+ggcyto_par_set(limits = "instrument")+geom_gate(g.Tcells)+scale_x_logicle()+scale_y_logicle()

ggcyto(gs_all[[3]],aes(x="CD3",y="CD19"), subset = "g.live")+geom_hex(bins = 512)+geom_stats(adjust = 1)+ggcyto_par_set(limits = "instrument")+geom_gate(g.Tcells)+scale_x_logicle()+scale_y_logicle()

# none T-cell gate
g.noT<-polygonGate("CD3"=c(-300,4000,4000,-300),"CD19"=c(-400,-400,3e5,3e5), filterId = "g.noT")
ggcyto(gs_all[[1]],aes(x="CD3",y="CD19"), subset = "g.live")+geom_hex(bins = 512)+ggcyto_par_set(limits = "instrument")+geom_gate(g.noT)+geom_stats()+scale_x_logicle()+scale_y_logicle()+geom_gate(g.Tcells)

ggcyto(gs_all[[3]],aes(x="CD3",y="CD19"), subset = "g.live")+geom_hex(bins = 512)+ggcyto_par_set(limits = "instrument")+geom_gate(g.noT)+geom_stats()+scale_x_logicle()+scale_y_logicle()+geom_gate(g.Tcells)

ggcyto(gs_all,aes(x="CD3",y="CD19"), subset = "g.live")+geom_hex(bins = 512)+ggcyto_par_set(limits = "instrument")+geom_gate(g.noT)+geom_stats()+scale_x_logicle()+scale_y_logicle()+geom_gate(g.Tcells)

ggsave("CD3_CD19_plot.png",height = 4000, width = 6000,units = "px", path = output_path)
gs_pop_add(gs_all, g.Tcells, parent="g.live")
gs_pop_add(gs_all, g.noT, parent="g.live")
recompute(gs_all)

# 4. plasma cell / ASC gate
g.ASC<-polygonGate("CD38"=c(4e6,4e6,9e4,3e5,6e5),"CD27"=c(4000,4e5,4e5,1e4,7000), filterId = "g.ASC")
ggcyto(gs_all[[1]],aes(x="CD38",y="CD27"), subset = "g.noT")+geom_hex(bins = 256)+ggcyto_par_set(limits = "instrument")+geom_gate(g.ASC)+scale_x_logicle()+scale_y_logicle()

ggcyto(gs_all[[3]],aes(x="CD38",y="CD27"), subset = "g.noT")+geom_hex(bins = 256)+ggcyto_par_set(limits = "instrument")+geom_gate(g.ASC)+scale_x_logicle()+scale_y_logicle()

ggcyto(gs_all,aes(x="CD38",y="CD27"), subset = "g.noT")+geom_hex(bins = 256)+ggcyto_par_set(limits = "instrument")+geom_gate(g.ASC)+scale_x_logicle()+scale_y_logicle()

ggsave("CD38_CD27_plot.png",height = 4000, width = 6000,units = "px", path = output_path)
gs_pop_add(gs_all, g.ASC, parent="g.noT")
recompute(gs_all)
```

```
# 5. B cell gate
g.Bcells<-polygonGate("CD19"=c(1e4,2e5,2e5,1e4),"CD20"=c(2e4,2e4,4e5,4e5), filterI
d = "g.Bcells")
ggcyto(gs_all[[1]],aes(x="CD19",y="CD20"), subset = "g.noT")+geom_hex(bins = 256)+
ggcyto_par_set(limits = "instrument")+geom_gate(g.Bcells)+scale_x_logicle()+scale_
y_logicle()

ggcyto(gs_all[[3]],aes(x="CD19",y="CD20"), subset = "g.noT")+geom_hex(bins = 256)+
ggcyto_par_set(limits = "instrument")+geom_gate(g.Bcells)+scale_x_logicle()+scale_
y_logicle()

ggcyto(gs_all,aes(x="CD19",y="CD20"), subset = "g.noT")+geom_hex(bins = 256)+ggcyt
o_par_set(limits = "instrument")+geom_gate(g.Bcells)+scale_x_logicle()+scale_y_log
icle()

ggsave("CD19_CD3_plot.png",height = 4000, width = 6000,units = "px", path = output
_path)
gs_pop_add(gs_all, g.Bcells, parent="g.noT")
recompute(gs_all)
```

Proceed with gating the control cell populations dependent on the maturity marker within the respective dataset. In this example:
For CD45: add gating for (CD14+)CD45- cells
For CD56: add gating for (CD16+)CD56+ cells and CD56- cells
For CD138: add gating for CD138+ cells and CD138- cells
For HLA-DR: add gating for (CD3+)HLA- cells
For Ki67: add gating for Ki67+ cells and Ki67- cells

```
print(all.set.marker)
# gate (CD14+)CD45- cells
g.live.CD14pos<-rectangleGate("CD45"=c(300,1e4),"CD3"=c(4e5,2500), filterId = "g.l
ive.CD14pos")
ggcyto(gs_all[[1]],aes(x="CD45",y="CD3"), subset = "g.live")+geom_hex(bins = 256)+
ggcyto_par_set(limits = "instrument")+geom_gate(g.live.CD14pos)+scale_x_logicle()+
scale_y_logicle()

ggcyto(gs_all[[3]],aes(x="CD45",y="CD3"), subset = "g.live")+geom_hex(bins = 256)+
ggcyto_par_set(limits = "instrument")+geom_gate(g.live.CD14pos)+scale_x_logicle()+
scale_y_logicle()

ggcyto(gs_all,aes(x="CD45",y="CD3"), subset = "g.live")+geom_hex(bins = 256)+ggcyt
o_par_set(limits = "instrument")+geom_gate(g.live.CD14pos)+scale_x_logicle()+scale
_y_logicle()

ggsave("CD45neg_plot.png",height = 4000, width = 6000,units = "px", path = output_
path)
gs_pop_add(gs_all, g.live.CD14pos, parent="g.live")

# gate CD138+ and CD138- cells
g.live.CD138neg<-rectangleGate("CD138"=c(1500,-100),"CD3"=c(4e5,25), filterId = "g
.live.CD138neg")
ggcyto(gs_all[[1]],aes(x="CD138",y="CD3"), subset = "g.live")+geom_hex(bins = 256)
+ggcyto_par_set(limits = "instrument")+geom_gate(g.live.CD138neg)+scale_x_logicle(
)+scale_y_logicle()

g.live.CD138pos<-rectangleGate("CD138"=c(2000,2e5),"CD3"=c(4e5,25), filterId = "g.
live.CD138pos")
```

```
ggcyto(gs_all[[3]],aes(x="CD138",y="CD3"), subset = "g.live")+geom_hex(bins = 256)
+ggcyto_par_set(limits = "instrument")+geom_gate(g.live.CD138neg)+geom_gate(g.live
.CD138pos)+scale_x_logicle()+scale_y_logicle()

ggcyto(gs_all,aes(x="CD138",y="CD3"), subset = "g.live")+geom_hex(bins = 256)+geom
_gate(g.live.CD138neg)+geom_gate(g.live.CD138pos)+scale_x_logicle()+scale_y_logicl
e()

ggsave("CD138_CD3_plot.png",height = 4000, width = 6000,units = "px", path = outpu
t_path)
gs_pop_add(gs_all, g.live.CD138neg, parent="g.live")
gs_pop_add(gs_all, g.live.CD138pos, parent="g.live")

# gate (CD3+)HLA- cells
g.T.HLAneg<-rectangleGate("CD3"=c(3000,1e5),"HLA.DR"=c(-300,1e3), filterId = "g.T.
HLAneg")
ggcyto(gs_all[[1]],aes(x="CD3",y="HLA.DR"), subset = "g.live")+geom_hex(bins = 256
)+ggcyto_par_set(limits = "instrument")+geom_gate(g.T.HLAneg)+scale_x_logicle()+sc
ale_y_logicle()

ggcyto(gs_all[[3]],aes(x="CD3",y="HLA.DR"), subset = "g.live")+geom_hex(bins = 256
)+ggcyto_par_set(limits = "instrument")+geom_gate(g.T.HLAneg)+scale_x_logicle()+sc
ale_y_logicle()

ggcyto(gs_all,aes(x="CD3",y="HLA.DR"), subset = "g.live")+geom_hex(bins = 256)+ggc
yto_par_set(limits = "instrument")+geom_gate(g.T.HLAneg)+scale_x_logicle()+scale_y
_logicle()

ggsave("CD3_HLA.DR_plot.png",height = 4000, width = 6000,units = "px", path = outp
ut_path)
gs_pop_add(gs_all, g.T.HLAneg, parent="g.live")

recompute(gs_all) #apply control populations


# gate CD56- cells
g.live.CD56neg<-rectangleGate("CD56"=c(-150,4e3),"CD3"=c(50,9e4), filterId = "g.li
ve.CD56neg")
ggcyto(gs_all[[1]],aes(x="CD56",y="CD3"), subset = "g.live")+geom_hex(bins = 256)+
ggcyto_par_set(limits = "instrument")+geom_gate(g.live.CD56neg)+scale_x_logicle()+
scale_y_logicle()

ggcyto(gs_all[[3]],aes(x="CD56",y="CD3"), subset = "g.live")+geom_hex(bins = 256)+
ggcyto_par_set(limits = "instrument")+geom_gate(g.live.CD56neg)+scale_x_logicle()+
scale_y_logicle()

ggcyto(gs_all,aes(x="CD56",y="CD3"), subset = "g.live")+geom_hex(bins = 256)+ggcyt
o_par_set(limits = "instrument")+geom_gate(g.live.CD56neg)+scale_x_logicle()+scale
_y_logicle()

ggsave("CD56_CD3_plot.png",height = 4000, width = 6000,units = "px", path = output
_path)
gs_pop_add(gs_all, g.live.CD56neg, parent="g.live")

recompute(gs_all) #apply control populations
```

```
# gate Ki67 cells
g.live.Ki67neg<-rectangleGate("Ki67"=c(100,5000),"CD3"=c(-200,1e5), filterId = "g.live.Ki67neg")

g.live.Ki67pos<-rectangleGate("Ki67"=c(8000,8e5),"CD3"=c(-200,1e5), filterId = "g.live.Ki67pos")
ggcyto(gs_all[[1]],aes(x="Ki67",y="CD3"), subset = "g.live")+geom_hex(bins = 256)+ggcyto_par_set(limits = "instrument")+geom_gate(g.live.Ki67neg)+geom_gate(g.live.Ki67pos)+scale_x_logicle()+scale_y_logicle()

ggcyto(gs_all,aes(x="Ki67",y="CD3"), subset = "g.live")+geom_hex(bins = 256)+ggcyto_par_set(limits = "instrument")+geom_gate(g.live.Ki67neg)+geom_gate(g.live.Ki67pos)+scale_x_logicle()+scale_y_logicle()

ggsave("Ki67_CD3_plot.png",height = 4000, width = 6000,units = "px", path = output_path)
gs_pop_add(gs_all, g.live.Ki67neg, parent="g.live")

gs_pop_add(gs_all, g.live.Ki67pos, parent="g.live")

recompute(gs_all) #apply control populations


# gate CD56+ cells
g.live.CD16CD56<-rectangleGate("CD56"=c(2e4,1e6),"CD3"=c(100,2e3), filterId = "g.live.CD16CD56")
ggcyto(gs_all[[1]],aes(x=" CD56",y=" CD3"), subset = "g.live")+geom_hex(bins = 256)+ggcyto_par_set(limits = "instrument")+geom_gate(g.live. CD16CD56)+scale_x_logicle()+scale_y_logicle()

ggcyto(gs_all[[3]],aes(x=" CD56",y=" CD3"), subset = "g.live")+geom_hex(bins = 256)+ggcyto_par_set(limits = "instrument")+geom_gate(g.live. CD16CD56)+scale_x_logicle()+scale_y_logicle()

ggcyto(gs_all,aes(x=" CD56",y=" CD3"), subset = "g.live")+geom_hex(bins = 256)+ggcyto_par_set(limits = "instrument")+geom_gate(g.live. CD16CD56)+scale_x_logicle()+scale_y_logicle()

ggsave("CD56_CD16_plot.png",height = 4000, width = 6000,units = "px", path = output_path)
gs_pop_add(gs_all, g.live.CD16CD56, parent="g.live")

recompute(gs_all) #apply control populations
```

Proceed with extracting expression values from the gated populations.

```
# defining a function to read out mean fluorescence intensities (MedFI)
pop.means <- function(gs_all){
  chnls <- colnames(gs_all)
  res <- colMeans(exprs(gs_all))
  names(res) <- chnls
  res
}

# write the MedFI values for all gated populations and all markers into a table
plot(gs_all)
```

```
all.pop<-c("g.live"
           ,"g.Tcells"
           ,"g.ASC"
           ,"g.Bcells"
           ,"g.live.CD14pos"
           ,"g.T.HLAneg"
           ,"g.live.CD138neg"
           ,"g.live.CD138pos"
           ,"g.live.CD56neg"
           ,"g.live.CD16CD56"
           ,"g.live.Ki67neg"
           ,"g.live.Ki67pos"
)
results_mean<-gs_pop_get_stats(gs_all, all.pop, type = pop.means)

# extract number of gated ASCs and combine it with the MedFI values
counts<-gs_pop_get_stats(gs_all, all.pop)
N.col.res_mean=ncol(results_mean)
results<-cbind(counts,results_mean[,3:N.col.res_mean])

# define number and position of relevant output data within the dataset (equal to
position within the all.pop object)
N.smpl=length(files)
N.pops=length(all.pop)
line.ASC=3
line.Bcells=4
line.Tcells=2
line.CD14=5
line.T.HLAneg=6
line.CD138neg=7
line.CD138pos=8
line.CD56neg=9
line.CD16CD56=10
line.Ki67pos=11
line.Ki67neg=12

# generate a dummy table for a standardized output table also including markers no
t included in the dataset
dummy_tab = data.frame(
  CD19 = matrix("NA",N.smpl),
  CD20 = matrix("NA",N.smpl),
  CD28 = matrix("NA",N.smpl),
  CD44 = matrix("NA",N.smpl),
  CD45 = matrix("NA",N.smpl),
  CD56 = matrix("NA",N.smpl),
  CD69 = matrix("NA",N.smpl),
  CD86 = matrix("NA",N.smpl),
  CD95 = matrix("NA",N.smpl),
  CD98 = matrix("NA",N.smpl),
  CD138 = matrix("NA",N.smpl),
  HLA.DR = matrix("NA",N.smpl),
  Ki67 = matrix("NA",N.smpl),
  Bcl.2 = matrix("NA",N.smpl),
  BAFFR = matrix("NA",N.smpl),
  BCMA = matrix("NA",N.smpl),
```

```
  TACI = matrix("NA",N.smpl)
)

# generate a new table with sample names and ASC counts
sampleID<-results[as.numeric(paste(line.ASC+N.pops*0:(N.smpl-1))),c(1,3)]
# calculate double normalized ASC expression values for each marker and add them t
o the table
# select/un-select required markers using '#'
{
  ##add CD19 (here this marker is added)
  sampleID<-cbind(sampleID,(results[as.numeric(paste(line.ASC+N.pops*0:(N.smpl-1))
),"CD19"]-results[as.numeric(paste(line.Tcells+N.pops*0:(N.smpl-1))),"CD19"])/(res
ults[as.numeric(paste(line.Bcells+N.pops*0:(N.smpl-1))),"CD19"]-results[as.numeric
(paste(line.Tcells+N.pops*0:(N.smpl-1))),"CD19"]))
  #sampleID<-cbind(sampleID,dummy_tab[,1])
  #names(sampleID)[3] <- "CD19"
  ##add CD20 (here this marker is added)
  sampleID<-cbind(sampleID,(results[as.numeric(paste(line.ASC+N.pops*0:(N.smpl-1))
),"CD20"]-results[as.numeric(paste(line.Tcells+N.pops*0:(N.smpl-1))),"CD20"])/(res
ults[as.numeric(paste(line.Bcells+N.pops*0:(N.smpl-1))),"CD20"]-results[as.numeric
(paste(line.Tcells+N.pops*0:(N.smpl-1))),"CD20"]))
  #sampleID<-cbind(sampleID,dummy_tab[,2])
  #names(sampleID)[4] <- "CD20"
  ##add CD28 (here this marker is skipped and NA input is used)
  #sampleID<-cbind(sampleID,(results[as.numeric(paste(line.ASC+N.pops*0:(N.smpl-1)
)),"CD28"]-results[as.numeric(paste(line.Bcells+N.pops*0:(N.smpl-1))),"CD28"])/(re
sults[as.numeric(paste(line.Tcells+N.pops*0:(N.smpl-1))),"CD28"]-results[as.numeri
c(paste(line.Bcells+N.pops*0:(N.smpl-1))),"CD28"]))
  sampleID<-cbind(sampleID,dummy_tab[,3])
  names(sampleID)[5] <- "CD28"
  ##add CD44 (here this marker is skipped and NA input is used)
  #sampleID<-cbind()
  sampleID<-cbind(sampleID,dummy_tab[,4])
  names(sampleID)[6] <- "CD44"
  ##add CD45 (here this marker is added)
  sampleID<-cbind(sampleID,(results[as.numeric(paste(line.ASC+N.pops*0:(N.smpl-1))
),"CD45"]-results[as.numeric(paste(line.CD14+N.pops*0:(N.smpl-1))),"CD45"])/(resul
ts[as.numeric(paste(line.Bcells+N.pops*0:(N.smpl-1))),"CD45"]-results[as.numeric(p
aste(line.CD14+N.pops*0:(N.smpl-1))),"CD45"]))
  #sampleID<-cbind(sampleID,dummy_tab[,5])
  #names(sampleID)[7] <- "CD45"
  ##add CD56 (here this marker is added)
  sampleID<-cbind(sampleID,(results[as.numeric(paste(line.ASC+N.pops*0:(N.smpl-1))
),"CD56"]-results[as.numeric(paste(line.CD56neg+N.pops*0:(N.smpl-1))),"CD56"])/(re
sults[as.numeric(paste(line.CD16CD56+N.pops*0:(N.smpl-1))),"CD56"]-results[as.nume
ric(paste(line.CD56neg +N.pops*0:(N.smpl-1))),"CD56"]))
  # sampleID<-cbind(sampleID,dummy_tab[,6])
  #names(sampleID)[7] <- "CD56"
  ##add CD69 (here this marker is skipped and NA input is used)
  #sampleID<-cbind(sampleID,(results[as.numeric(paste(line.ASC+N.pops*0:(N.smpl-1)
)),"CD69"]-results[as.numeric(paste(line.CD69neg+N.pops*0:(N.smpl-1))),"CD69"])/(r
esults[as.numeric(paste(line.CD14CD69+N.pops*0:(N.smpl-1))),"CD69"]-results[as.num
eric(paste(line.CD69neg+N.pops*0:(N.smpl-1))),"CD69"]))
  sampleID<-cbind(sampleID,dummy_tab[,7])
  names(sampleID)[9] <- "CD69"
```

```
  ##add CD86 (here this marker is skipped and NA input is used)
  #sampleID<-cbind(sampleID,(results[as.numeric(paste(line.ASC+N.pops*0:(N.smpl-1)
)),"CD86"]-results[as.numeric(paste(line.CD86neg+N.pops*0:(N.smpl-1))),"CD86"])/(r
esults[as.numeric(paste(line.Bcells+N.pops*0:(N.smpl-1))),"CD86"]-results[as.numer
ic(paste(line.CD86neg+N.pops*0:(N.smpl-1))),"CD86"]))
  sampleID<-cbind(sampleID,dummy_tab[,8])
  names(sampleID)[10] <- "CD86"
  ##add CD95 (here this marker is skipped and NA input is used)
  #sampleID<-cbind(sampleID,(results[as.numeric(paste(line.ASC+N.pops*0:(N.smpl-1)
)),"CD95"]-results[as.numeric(paste(line.CD95neg+N.pops*0:(N.smpl-1))),"CD95"])/(r
esults[as.numeric(paste(line.Bcells+N.pops*0:(N.smpl-1))),"CD95"]-results[as.numer
ic(paste(line.CD95neg+N.pops*0:(N.smpl-1))),"CD95"]))
  sampleID<-cbind(sampleID,dummy_tab[,9])
  names(sampleID)[11] <- "CD95"
  ##add CD98 (here this marker is skipped and NA input is used)
  #sampleID<-cbind()
  sampleID<-cbind(sampleID,dummy_tab[,10])
  names(sampleID)[12] <- "CD98"
  ##add CD138 (here this marker is added)
  sampleID<-cbind(sampleID,(results[as.numeric(paste(line.ASC+N.pops*0:(N.smpl-1))
),"CD138"]-results[as.numeric(paste(line.CD138neg+N.pops*0:(N.smpl-1))),"CD138"])/
(results[as.numeric(paste(line.CD138pos+N.pops*0:(N.smpl-1))),"CD138"]-results[as.
numeric(paste(line.CD138neg+N.pops*0:(N.smpl-1))),"CD138"]))
  #sampleID<-cbind(sampleID,dummy_tab[,11])
  #names(sampleID)[13] <- "CD138"
  ##add HLA.DR (here this marker is added)
  sampleID<-cbind(sampleID,(results[as.numeric(paste(line.ASC+N.pops*0:(N.smpl-1))
),"HLA.DR"]-results[as.numeric(paste(line.T.HLAneg+N.pops*0:(N.smpl-1))),"HLA.DR"]
)/(results[as.numeric(paste(line.Bcells+N.pops*0:(N.smpl-1))),"HLA.DR"]-results[as
.numeric(paste(line.T.HLAneg+N.pops*0:(N.smpl-1))),"HLA.DR"]))
  #sampleID<-cbind(sampleID,dummy_tab[,12])
  #names(sampleID)[14] <- "HLA.DR"
  ##add Ki67 (here this marker is added)
  sampleID<-cbind(sampleID,(results[as.numeric(paste(line.ASC+N.pops*0:(N.smpl-1))
),"Ki67"]-results[as.numeric(paste(line.Ki67neg+N.pops*0:(N.smpl-1))),"Ki67"])/(re
sults[as.numeric(paste(line.Ki67pos+N.pops*0:(N.smpl-1))),"Ki67"]-results[as.numer
ic(paste(line.Ki67neg+N.pops*0:(N.smpl-1))),"Ki67"]))
  #sampleID<-cbind(sampleID,dummy_tab[,13])
  #names(sampleID)[14] <- "Ki67"
  ##add Bcl.2 (here this marker is skipped and NA input is used)
  #sampleID<-cbind(sampleID,(results[as.numeric(paste(line.ASC+N.pops*0:(N.smpl-1)
)),"Bcl.2"]-results[as.numeric(paste(line.BCL2neg+N.pops*0:(N.smpl-1))),"Bcl.2"])/
(results[as.numeric(paste(line.Bcells+N.pops*0:(N.smpl-1))),"Bcl.2"]-results[as.nu
meric(paste(line.BCL2neg+N.pops*0:(N.smpl-1))),"Bcl.2"]))
  sampleID<-cbind(sampleID,dummy_tab[,14])
  names(sampleID)[16] <- "Bcl.2"
  ##add BAFFR (here this marker is skipped and NA input is used)
  #sampleID<-cbind()
  sampleID<-cbind(sampleID,dummy_tab[,15])
  names(sampleID)[17] <- "BAFFR"
  ##add BCMA (here this marker is skipped and NA input is used)
  #sampleID<-cbind(sampleID,(results[as.numeric(paste(line.ASC+N.pops*0:(N.smpl-1)
)),"BCMA"]-results[as.numeric(paste(line.BCMAneg+N.pops*0:(N.smpl-1))),"BCMA"])/(r
esults[as.numeric(paste(line.Bcells+N.pops*0:(N.smpl-1))),"BCMA"]-results[as.numer
ic(paste(line.BCMAneg+N.pops*0:(N.smpl-1))),"BCMA"]))
```

```
  sampleID<-cbind(sampleID,dummy_tab[,16])
  names(sampleID)[18] <- "BCMA"
  ##add TACI (here this marker is skipped and NA input is used)
  #sampleID<-cbind(sampleID,(results[as.numeric(paste(line.ASC+N.pops*0:(N.smpl-1)
)),"TACI"]-results[as.numeric(paste(line.TACIneg+N.pops*0:(N.smpl-1))),"TACI"])/(r
esults[as.numeric(paste(line.Bcells+N.pops*0:(N.smpl-1))),"TACI"]-results[as.numer
ic(paste(line.TACIneg+N.pops*0:(N.smpl-1))),"TACI"]))
  sampleID<-cbind(sampleID,dummy_tab[,17])
  names(sampleID)[19] <- "TACI"
}

# As quality control of the results: write mean, maximal and minimal value of each
marker into a new table
{
  # select/un-select required markers using '#'
  QC_sampleID = data.frame(QC=c("mean","max","min"))
  QC_sampleID<-cbind(QC_sampleID, CD19=c(colMeans(sampleID[,3],na.rm = TRUE),max(s
ampleID[,3],na.rm = TRUE),min(sampleID[,3],na.rm = TRUE)))
  QC_sampleID<-cbind(QC_sampleID, CD20=c(colMeans(sampleID[,4],na.rm = TRUE),max(s
ampleID[,4],na.rm = TRUE),min(sampleID[,4],na.rm = TRUE)))
  #QC_sampleID<-cbind(QC_sampleID, CD28=c(colMeans(sampleID[,5],na.rm = TRUE),max(
sampleID[,5],na.rm = TRUE),min(sampleID[,5],na.rm = TRUE)))
  #QC_sampleID<-cbind(QC_sampleID, CD44=c(colMeans(sampleID[,6],na.rm = TRUE),max(
sampleID[,6],na.rm = TRUE),min(sampleID[,6],na.rm = TRUE)))
  QC_sampleID<-cbind(QC_sampleID, CD45=c(colMeans(sampleID[,7],na.rm = TRUE),max(s
ampleID[,7],na.rm = TRUE),min(sampleID[,7],na.rm = TRUE)))
  QC_sampleID<-cbind(QC_sampleID, CD56=c(colMeans(sampleID[,8],na.rm = TRUE),max(s
ampleID[,8],na.rm = TRUE),min(sampleID[,8],na.rm = TRUE)))
  #QC_sampleID<-cbind(QC_sampleID, CD69=c(colMeans(sampleID[,9],na.rm = TRUE),max(
sampleID[,9],na.rm = TRUE),min(sampleID[,9],na.rm = TRUE)))
  #QC_sampleID<-cbind(QC_sampleID, CD86=c(colMeans(sampleID[,10],na.rm = TRUE),max
(sampleID[,10],na.rm = TRUE),min(sampleID[,10],na.rm = TRUE)))
  #QC_sampleID<-cbind(QC_sampleID, CD95=c(colMeans(sampleID[,11],na.rm = TRUE),max
(sampleID[,11],na.rm = TRUE),min(sampleID[,11],na.rm = TRUE)))
  #QC_sampleID<-cbind(QC_sampleID, CD98=c(colMeans(sampleID[,12],na.rm = TRUE),max
(sampleID[,12],na.rm = TRUE),min(sampleID[,12],na.rm = TRUE)))
  QC_sampleID<-cbind(QC_sampleID, CD138=c(colMeans(sampleID[,13],na.rm = TRUE),max
(sampleID[,13],na.rm = TRUE),min(sampleID[,13],na.rm = TRUE)))
  QC_sampleID<-cbind(QC_sampleID, HLA.DR=c(colMeans(sampleID[,14],na.rm = TRUE),ma
x(sampleID[,14],na.rm = TRUE),min(sampleID[,14],na.rm = TRUE)))
  QC_sampleID<-cbind(QC_sampleID, Ki67=c(colMeans(sampleID[,15],na.rm = TRUE),max(
sampleID[,15],na.rm = TRUE),min(sampleID[,15],na.rm = TRUE)))
  #QC_sampleID<-cbind(QC_sampleID, Bcl.2=c(colMeans(sampleID[,16],na.rm = TRUE),ma
x(sampleID[,16],na.rm = TRUE),min(sampleID[,16],na.rm = TRUE)))
  #QC_sampleID<-cbind(QC_sampleID, BAFFR=c(colMeans(sampleID[,17],na.rm = TRUE),ma
x(sampleID[,17],na.rm = TRUE),min(sampleID[,17],na.rm = TRUE)))
  #QC_sampleID<-cbind(QC_sampleID, BCMA=c(colMeans(sampleID[,18],na.rm = TRUE),max
(sampleID[,18],na.rm = TRUE),min(sampleID[,18],na.rm = TRUE)))
  #QC_sampleID<-cbind(QC_sampleID, TACI=c(colMeans(sampleID[,19],na.rm = TRUE),max
(sampleID[,19],na.rm = TRUE),min(sampleID[,19],na.rm = TRUE)))
}

# export all results for further quality control and subsequent data processing
write_xlsx(QC_sampleID, path = paste(output_path,"/QC_results.xlsx", sep = ""))
```

```
write_xlsx(results, path = paste(output_path,"/results.xlsx", sep = ""))
write_xlsx(sampleID, path = paste(output_path,"/final_results.xlsx", sep = ""))
```

Generate overlay plots of each marker with ASCs, positive and negative control populations for each individual sample as visual quality control.

```
# plot overlays
for(n in 1:length(files)) {
  if (sampleID[n,2] == 0) {next}
  gc()
  ggcyto(gs_all[[n]],aes(x="CD19",y="CD3"), subset = "g.ASC")+scale_fill_gradient(
trans = "sqrt", high = "black")+geom_overlay(data="g.Tcells", size=0.01, color="ye
llowgreen")+geom_overlay(data="g.Bcells", size=0.01,color="tomato3")+geom_hex(bins
=64)+ggcyto_par_set(limits = "instrument")+scale_x_logicle()+scale_y_logicle()+the
me(axis.text.x=element_blank(),axis.text.y=element_blank())
  ggsave(filename = paste("CD19_overlay_",n,".png", sep = ""), height = 800, width
= 1000,units = "px", path = output_path)
  ggcyto(gs_all[[n]],aes(x="CD20",y="CD3"), subset = "g.ASC")+scale_fill_gradient(
trans = "sqrt", high = "black")+geom_overlay(data="g.Tcells", size=0.01, color="ye
llowgreen")+geom_overlay(data="g.Bcells", size=0.01,color="tomato3")+geom_hex(bins
=64)+ggcyto_par_set(limits = "instrument")+scale_x_logicle()+scale_y_logicle()+the
me(axis.text.x=element_blank(),axis.text.y=element_blank())
  ggsave(filename = paste("CD20_overlay_",n,".png", sep = ""), height = 800, width
= 1000,units = "px", path = output_path)
  ggcyto(gs_all[[n]],aes(x="CD28",y="CD3"), subset = "g.ASC")+scale_fill_gradient(
trans = "sqrt", high = "black")+geom_overlay(data="g.Bcells", size=0.01, color="ye
llowgreen")+geom_overlay(data="g.Tcells", size=0.01,color="tomato3")+geom_hex(bins
=64)+ggcyto_par_set(limits = "instrument")+scale_x_logicle()+scale_y_logicle()+the
me(axis.text.x=element_blank(),axis.text.y=element_blank())
  ggsave(filename = paste("CD28_overlay_",n,".png", sep = ""), height = 800, width
= 1000,units = "px", path = output_path)
  ggcyto(gs_all[[n]],aes(x="CD45",y="CD19"), subset = "g.ASC")+scale_fill_gradient
(trans = "sqrt", high = "black")+geom_overlay(data="g.live.CD14pos", size=0.01, co
lor="yellowgreen")+geom_overlay(data="g.Bcells", size=0.01,color="tomato3")+geom_h
ex(bins=64)+ggcyto_par_set(limits = "instrument")+scale_x_logicle()+scale_y_logicl
e()+theme(axis.text.x=element_blank(),axis.text.y=element_blank())
  ggsave(filename = paste("CD45_overlay_",n,".png", sep = ""), height = 800, width
= 1000,units = "px", path = output_path)
  ggcyto(gs_all[[n]],aes(x="CD56",y="CD19"), subset = "g.ASC")+scale_fill_gradient
(trans = "sqrt", high = "black")+geom_overlay(data="g.live.CD56neg", size=0.01, co
lor="yellowgreen")+geom_overlay(data="g.live.CD16CD56", size=0.01,color="tomato3")
+geom_hex(bins=64)+ggcyto_par_set(limits = "instrument")+scale_x_logicle()+scale_y
_logicle()+theme(axis.text.x=element_blank(),axis.text.y=element_blank())
  ggsave(filename = paste("CD56_overlay_",n,".png", sep = ""), height = 800, width
= 1000,units = "px", path = output_path)
  ggcyto(gs_all[[n]],aes(x="CD138",y="CD19"), subset = "g.ASC")+scale_fill_gradien
t(trans = "sqrt", high = "black")+geom_overlay(data="g.live.CD138neg", size=0.01,
color="yellowgreen")+geom_overlay(data="g.live.CD138pos", size=0.01,color="tomato3
")+geom_hex(binwidth=c(0.03,0.03))+ggcyto_par_set(limits = "instrument")+scale_x_l
ogicle()+scale_y_logicle()+theme(axis.text.x=element_blank(),axis.text.y=element_b
lank())
  ggsave(filename = paste("CD138_overlay_",n,".png", sep = ""), height = 800, widt
h = 1000,units = "px", path = output_path)
  ggcyto(gs_all[[n]],aes(x="HLA.DR",y="CD19"), subset = "g.ASC")+scale_fill_gradie
nt(trans = "sqrt", high = "black")+geom_overlay(data="g.T.HLAneg", size=0.01, colo
r="yellowgreen")+geom_overlay(data="g.Bcells", size=0.01,color="tomato3")+geom_hex
```

```
(binwidth=c(0.03,0.03))+ggcyto_par_set(limits = "instrument")+scale_x_logicle()+scale_y_logicle()+theme(axis.text.x=element_blank(),axis.text.y=element_blank())
  ggsave(filename = paste("HLA_overlay_",n,".png", sep = ""), height = 800, width = 1000,units = "px", path = output_path)
  ggcyto(gs_all[[n]],aes(x="Ki67",y="CD19"), subset = "g.ASC")+scale_fill_gradient(trans = "sqrt", high = "black")+geom_overlay(data="g.live.Ki67neg", size=0.01, color="yellowgreen")+geom_overlay(data="g.live.Ki67pos", size=0.01,color="tomato3")+geom_hex(bins=64)+ggcyto_par_set(limits = "instrument")+scale_x_logicle()+scale_y_logicle()+theme(axis.text.x=element_blank(),axis.text.y=element_blank())
  ggsave(filename = paste("Ki67_overlay_",n,".png", sep = ""), height = 800, width = 1000,units = "px", path = output_path)
}
```

### *Preparation of data for subsequent input into the setup of the random forest prediction model*

After extraction of normalized expression values from all included datasets, the total data was processed to comply with the requirements of the random forest model training in the next step.
This includes combining the normalized MedFI results from the analysis above with the sample meta-data, standardizing the reporting of various parameters like sex, race and dataset type, with subsequent filtering of the data for those datasets containing samples from controlled infection of vaccination kinetics.

```
# load the libraries required for this project step
library(readxl)

# import output of cytometry analysis
parent_folder <- "…/.ASC_ME_project/.new_output" #directory with cytometry output
subfolders <- list.dirs(parent_folder, full.names = TRUE, recursive = FALSE) #bind folder paths into a list

# loop through the subfolders and read in each output file containing the normalized ASC expression values (final_results.xlsx)
all_data <- lapply(subfolders, function(folder) {
  file_path <- file.path(folder, "final_results.xlsx")

  if (file.exists(file_path)) {
    df <- read_excel(file_path)
    df[,c(3:15)] <- lapply(df[,c(3:15)], as.numeric)
    df$subfolder <- basename(folder)  #add subfolder name as a new column
    return(df)
  } else {
    message("No final_results.xlsx found in: ", folder)
    return(NULL)
  }
}) %>%
  bind_rows()  #combine all

# import the meta data of all datasets stored in txt- and xlsx- files
{
  data_folder <- "…/.ASC_ME_project/.sample_data"
  txt_files <- list.files(data_folder, pattern = "\\.txt$", full.names = TRUE)
  xlsx_files<- list.files(data_folder, pattern = "\\.xlsx$", full.names = TRUE)
```

```
  # Loop through and read each txt- and xlsx-file
  sample_annotations <- lapply(txt_files, function(file) {
    df <- read.delim(file, header = TRUE, sep = "\t", stringsAsFactors = FALSE)

    df$Planned.Visit.Name <- lapply(df$Planned.Visit.Name, as.character)
    # Add a column with the filename (without extension)
    df$source_file <- tools::file_path_sans_ext(basename(file))

    return(df)
  }) %>%
    bind_rows()

  colnames(sample_annotations)[colnames(sample_annotations)=="File.Name"] <- "samp
le"

  xlsx_annotations <- lapply(xlsx_files, function(file) {
    df <- read_excel(file)
    df$Subject.Age <- as.numeric(df$Subject.Age)
    df$Study.Time.Collected <- as.numeric(df$Study.Time.Collected)
    df$Subject.Accession <- as.character(df$Subject.Accession)
    df$source_file <- tools::file_path_sans_ext(basename(file))

    return(df)
  }) %>%
    bind_rows()

  all_annotations<-rbind(sample_annotations[,c(1,3:5,7,10,13:15,20,22,30,38:41)],x
lsx_annotations)
  }
```

Merge the normalized expression values and the accompanying meta-data using the unique sample name (left_join by sample). Data used for the setup of the random forest prediction model was filtered to apply thresholds for age >= 10 and an ASC count of >= 50. Samples with a follow-up time (days post immune intervention; dpi) of more than 180 days were excluded to lower the risk that new vaccination or infections during the follow-up introduce noise to the training data.

```
# joining both dataframes together while also streamline Gender/Sex, Race/Ethnicit
y and cytometry type
joined_data<-left_join(all_data, all_annotations, by = "sample") %>%
  mutate(across(Gender, ~ gsub("Not Specified", "NA",.))) %>%
  mutate(across(Gender, ~ gsub("Female", "female",.))) %>%
  mutate(across(Gender, ~ gsub("Male", "male",.))) %>%
  mutate(across(Race, ~ gsub("Other", "NA/other",.))) %>%
  mutate(across(Race, ~ gsub("American Indian or Alaska Native", "NA/other",.))) %
>%
  mutate(across(Race, ~ gsub("Not Specified", "NA/other",.))) %>%
  mutate(across(Race, ~ gsub("Native Hawaiian or NA/other Pacific Islander", "NA/o
ther",.))) %>%
  mutate(across(Race, ~ gsub("Unknown", "NA/other",.))) %>%
  mutate(Race   = ifelse(is.na(Race), "NA/other", Race), Gender = ifelse(is.na(Gen
der), "NA/other", Gender)) %>%
  mutate(across(File.Detail, ~ gsub("flow", "flow_cyto",.))) %>%
  mutate(across(File.Detail, ~ gsub("Flow cytometry result", "flow_cyto",.))) %>%
  mutate(across(File.Detail, ~ gsub("CyTOF result", "mass_cyto",.))) %>%
```

```
  mutate(across(File.Detail, ~ gsub("CyTOF", "mass_cyto",.)))

# filter for those datasets that contain kinetic data from controlled infections a
nd vaccinations suitable for model training and validation
filtered_kinetic_data<-joined_data %>% filter(count>49) %>% filter(Subject.Age>9.9
999) %>%
  filter((source_file=="AID_UMCG")|(source_file=="CER_UMCG")|(source_file=="SDY108
6_sample")|(source_file=="SDY1288_sample")|
           (source_file=="SDY144_sample")|(source_file=="SDY1669_sample")|(source_
file=="SDY1734_sample")|
           (source_file=="SDY224_sample")|(source_file=="SDY364_sample")|(source_f
ile=="SDY387_sample")|
           (source_file=="SDY522_sample")|(source_file=="SDY272_sample")|(source_f
ile=="SDY984_sample")|
           (source_file=="SDY80_sample")|(source_file=="SDY180_sample")|(source_fi
le=="SDY296_sample")|
           (source_file=="SDY301_sample")|(source_file=="SDY819_sample")|(source_f
ile=="ZY77")|(source_file=="SDY1397_sample")|
           (source_file=="SDY368_sample")|(source_file=="SDY788_sample")|(source_f
ile=="SDY34_sample")|
           (source_file=="SDY648_sample")|(source_file=="SDY739_sample"))

# streamline sample annotations for kinetic information and filter for it
filtered_kinetic_data<-filtered_kinetic_data %>%
  mutate(across(ARM.Name, ~ gsub("Vax002_group_A", "HC_Vac",.))) %>%
  mutate(across(ARM.Name, ~ gsub("Vax002_group_B", "HC_Vac",.))) %>%
  mutate(across(ARM.Name, ~ gsub("CHIKV patient", "HC_Inf",.))) %>%
  mutate(across(ARM.Name, ~ gsub("Infected individual", "HC_Inf",.))) %>%
  mutate(across(ARM.Name, ~ gsub("TIV Vaccine", "HC_Vac",.))) %>%
  mutate(across(ARM.Name, ~ gsub("Emory cohort", "HC_Inf",.))) %>%
  mutate(across(ARM.Name, ~ gsub("Hong Kong cohort", "HC_Inf",.))) %>%
  mutate(across(ARM.Name, ~ gsub("Africans", "HC_Inf",.))) %>%
  mutate(across(ARM.Name, ~ gsub("Europeans", "HC_Inf",.))) %>%
  mutate(across(ARM.Name, ~ gsub("Study group 1 Pneunomax23", "HC_Vac",.))) %>%
  mutate(across(ARM.Name, ~ gsub("Study group 1 Saline", "excl",.))) %>%
  mutate(across(ARM.Name, ~ gsub("Study group 1 2009-2010 Fluzone", "HC_Vac",.)))
%>%
  mutate(across(ARM.Name, ~ gsub("TIV 2010", "HC_Vac",.))) %>%
  mutate(across(ARM.Name, ~ gsub("Young", "HC_Vac",.))) %>%
  mutate(across(ARM.Name, ~ gsub("Aged", "HC_Vac",.))) %>%
  mutate(across(ARM.Name, ~ gsub("AIRFV 2011-12", "HC_Vac",.))) %>%
  mutate(across(ARM.Name, ~ gsub("AIRFV 2012-13", "HC_Vac",.))) %>%
  mutate(across(ARM.Name, ~ gsub("Healthy Controls", "HC_Vac",.))) %>%
  mutate(across(ARM.Name, ~ gsub("6 month post-transplant subjects receiving triva
lent influenza vaccine", "TP_Vac",.))) %>%
  mutate(across(ARM.Name, ~ gsub("NCH-2012-13", "HC_Vac",.))) %>%
  mutate(across(ARM.Name, ~ gsub("NCH-2013-14", "HC_Vac",.))) %>%
  mutate(across(ARM.Name, ~ gsub("NCH-2010-11", "HC_Vac",.))) %>%
  mutate(across(ARM.Name, ~ gsub("LAIV Vaccine", "HC_Vac",.))) %>%
  mutate(across(ARM.Name, ~ gsub("Immune response to Influenza vaccination in aged
populations - Year 3", "HC_Vac",.))) %>%
  mutate(across(ARM.Name, ~ gsub("Immune response to Influenza vaccination in aged
populations - Year 4", "HC_Vac",.))) %>%
  mutate(across(ARM.Name, ~ gsub("control", "HC",.))) %>%
  mutate(across(ARM.Name, ~ gsub("Non-responder", "HC_Inf",.))) %>%
```

```
  mutate(across(ARM.Name, ~ gsub("Responder", "HC_Inf",.))) %>%
  mutate(across(ARM.Name, ~ gsub("Cohort2", "HC_Vac",.))) %>%
  mutate(across(ARM.Name, ~ gsub("Immune response to Influenza vaccination in aged
populations - Year 5", "HC_Vac",.))) %>%
  mutate(across(ARM.Name, ~ gsub("elderly", "HC_Vac",.))) %>%
  mutate(across(ARM.Name, ~ gsub("young", "HC_Vac",.))) %>%
  mutate(across(ARM.Name, ~ gsub("uncomplicated", "HC",.))) %>%
  mutate(across(ARM.Name, ~ gsub("infections", "HC_Inf",.))) %>%
  mutate(across(ARM.Name, ~ gsub("inf_relapse", "HC_Inf",.)))

filtered_kinetic_data<-filtered_kinetic_data %>%
  filter((ARM.Name=="HC_Vac")|(ARM.Name=="HC")|(ARM.Name=="HC_Inf")) %>%
  filter(Study.Time.Collected<181) #data with dpi>180 are excluded
```

Using the following function all available kinetic data (from controlled infection and vaccination studies) is randomly split into a training and a validation data subset. Data splitting was conducted along the following criteria:

1) Each dataset is exclusively allocated to training or validation subset to avoid compromising the integrity of external validation.
2) Around 75% training data and 25% validation data, with a tolerance of 2%
3) Equal distribution of the mean follow-up time of samples (days pasted since the beginning of an immune reaction) was set as primary parameter to choose the best split.
4) Equal distribution of Sex, Race and Age was included with a lower priority (0.2x weight compared to follow-up time).

The split that fit those criteria the best was chosen among 5000 iterations.

```
split_by_sourcefile <- function(filtered_kinetic_data,
                                source_col = "source_file",
                                cat_cols = c("Race", "Gender"),
                                num_cols = c("Subject.Age"),
                                balance_col = "Study.Time.Collected",  # main vari
able to balance
                                train_frac = 0.75,
                                iterations = 5000,
                                tol = 0.02,
                                seed = NULL) {
  library(dplyr)
  if (!is.null(seed)) set.seed(seed)

  df <- filtered_kinetic_data
  total_n <- nrow(df)
  target <- train_frac * total_n

  # summarize counts per source file
  file_summary <- df %>%
    group_by_at(source_col) %>%
    summarise(n = n(), .groups = "drop")
  files <- file_summary[[source_col]]

  best_score <- Inf
  best_train_files <- NULL
  valid_candidates <- 0L

  for (i in seq_len(iterations)) {
```

```
    perm <- sample(files)                                  # random order of files
    perm_counts <- file_summary$n[match(perm, files)]    # counts in that order
    cum_counts <- cumsum(perm_counts)
    k <- which.min(abs(cum_counts - target))             # choose cut closest to t
arget
    n_train <- cum_counts[k]

    if (abs(n_train - target) > tol * total_n) next

    train_files <- perm[1:k]
    train_df <- df[df[[source_col]] %in% train_files, , drop = FALSE]
    val_df   <- df[!df[[source_col]] %in% train_files, , drop = FALSE]

    ### Main balance: Study.Time.Collected
    train_bal <- suppressWarnings(as.numeric(train_df[[balance_col]]))
    val_bal   <- suppressWarnings(as.numeric(val_df[[balance_col]]))
    train_bal <- train_bal[!is.na(train_bal)]
    val_bal   <- val_bal[!is.na(val_bal)]

    if (length(train_bal) < 2 || length(val_bal) < 2) {
      main_score <- 0
    } else {
      pooled_sd <- sqrt(((length(train_bal)-1)*var(train_bal) + (length(val_bal)-1
)*var(val_bal)) /
                          (length(train_bal) + length(val_bal) - 2))
      if (is.na(pooled_sd) || pooled_sd == 0) pooled_sd <- 1
      main_score <- abs(mean(train_bal) - mean(val_bal)) / pooled_sd +
        abs(sd(train_bal) - sd(val_bal)) / pooled_sd
    }

    ### Secondary balance: Race, Gender, Age
    # categorical imbalance
    total_cat_score <- 0
    for (cc in cat_cols) {
      levs <- union(unique(train_df[[cc]]), unique(val_df[[cc]]))
      p_train <- prop.table(table(factor(train_df[[cc]], levels = levs)))
      p_val   <- prop.table(table(factor(val_df[[cc]],   levels = levs)))
      total_cat_score <- total_cat_score + sum(abs(as.numeric(p_train) - as.numeri
c(p_val)))
    }

    # numeric imbalance for Age
    total_num_score <- 0
    for (nc in num_cols) {
      train_num <- suppressWarnings(as.numeric(train_df[[nc]]))
      val_num   <- suppressWarnings(as.numeric(val_df[[nc]]))
      train_num <- train_num[!is.na(train_num)]
      val_num   <- val_num[!is.na(val_num)]
      if (length(train_num) >= 2 && length(val_num) >= 2) {
        pooled_sd <- sqrt(((length(train_num)-1)*var(train_num) + (length(val_num)
-1)*var(val_num)) /
                            (length(train_num) + length(val_num) - 2))
        if (is.na(pooled_sd) || pooled_sd == 0) pooled_sd <- 1
        total_num_score <- total_num_score +
          abs(mean(train_num) - mean(val_num)) / pooled_sd
```

```
      }
    }

    # penalty for deviating from exact train fraction
    sample_penalty <- abs(n_train/total_n - train_frac)

    ### Weighted total score
    score <- (main_score * 5) +          # strong weight on Study.Time.Collected
      (total_cat_score * 1) +      # lighter weight on Race & Gender
      (total_num_score * 1) +      # lighter weight on Age
      (sample_penalty * 10)

    if (score < best_score) {
      best_score <- score
      best_train_files <- train_files
    }
    valid_candidates <- valid_candidates + 1L
  }

  if (is.null(best_train_files)) {
    stop("No valid split found within tolerance. Try increasing `tol` or `iteratio
ns`.")
  }

  train_df <- df[df[[source_col]] %in% best_train_files, , drop = FALSE]
  val_df   <- df[!df[[source_col]] %in% best_train_files, , drop = FALSE]
  assignment <- data.frame(
    source_file = files,
    assigned = ifelse(files %in% best_train_files, "train", "val"),
    n = file_summary$n,
    stringsAsFactors = FALSE
  )

  cat("Split chosen:\n")
  cat(" - train samples:", nrow(train_df), "\n")
  cat(" - val   samples:", nrow(val_df), "\n")
  cat(" - achieved train fraction:", round(nrow(train_df)/total_n, 4), "\n")
  cat(" - best score:", round(best_score, 4), "\n")
  cat(" - valid candidates evaluated:", valid_candidates, " (of", iterations, ")\n
\n")

  return(list(train = train_df, val = val_df,
              assignment = assignment, score = best_score))
}
```

Use the above defined function to split the kinetic data into training and validation subsets:

```
# fix seed for reproducable outcome
res <- split_by_sourcefile(filtered_kinetic_data,
                           source_col = "source_file",
                           cat_cols = c("Race","Gender"),
                           num_cols = c("Subject.Age"),
                           balance_col = "Study.Time.Collected",
                           train_frac = 0.75,
                           iterations = 10000,
                           tol = 0.05,
```

```
                           seed = 557)
# write separate tables for training and validation subsets
train_df <- res$train
val_df   <- res$val
```

### *Setup for the maturity prediction model using random forest*

Normalized expression values of potential antibody-secreting cell maturity markers were used to set up a prediction model using random forest-based machine learning, since random forest models are suitable for continuous outcomes, resistant to overfitting and cope well with non-linear input variables.

Within the training subset, total of 795 samples from 17 datasets (Figure 1) from controlled infection and vaccination of healthy human individuals contained annotations of the day post immune-intervention (DPI) ranging from five to 180 days (i.e. kinetic samples). An additional 269 samples from 6 datasets were used for external validation. Based on the training set, a random forest prediction model was trained on predicting the DPI-timepoint of a sample based on various potential antibody-secreting cell maturity markers, namely CD19, CD20, CD28, CD45, CD56, CD138, HLA.DR, and Ki67 as final predictor setup, by using the following steps:

1) define the data used for setting up the model training. This includes a cut-off for the allowed normalized expression values within the limits of -2 and 5, since those extreme out-of-range values indicate insufficient normalization of the respective marker within a sample. This might be caused by variation in control populations or problems during staining or acquisition of individual samples.

```
library(randomForest)
library(caret)
library(mice)
library(gtools)
library(VIM)
library(randomForestExplainer)
library(tidyverse)
library(skimr)
library(viridis)
library(dunn.test)
library(gridExtra)

# data not required for the model setup are removed and columns are renamed
data <- train_df %>%
  select(-c(CD98,Bcl.2,BAFFR,BCMA,TACI,CD44,CD95,CD69,CD86,subfolder,V2,Race,Race.
Specify,Ethnicity,ARM.Accession,Expsample.Accession,Original.File.Name)) %>%
  filter(!(sample=="NA")) %>%
  rename(dpi = Study.Time.Collected) %>%
  rename(group = ARM.Name) %>%
  rename(datatype = File.Detail) %>%
  rename(Age = Subject.Age) %>%
  rename(StudyID = Study.Accession) %>%
  rename(SubjectID = Subject.Accession) %>%
  rename(organ = Biosample.Type) %>%
  mutate(across(3:10, ~ ifelse(.x < -2 | .x > 5, NA, .x)))
```

2) Missing maturity markers are imputed using the mice package with the random forest method for 20 iterations yielding a bundle of 20 imputed output tables.

```r
# separate parameters taking part in the imputation and those that should not infl
uence the imputation
dataformerging <- data %>% #parameters excluded from imputation
  select(sample,count,source_file,SubjectID)
dataforimp <- data %>% #parameters considered during imputation
  select(-c(count,source_file,SubjectID))
# running the data imputation
iter=20
imp2025 <- mice(dataforimp, method = "rf", m = iter, seed = 557)

# merge all imputed data
data.imputed <- list()
for(m in 1:20){
  data.imputed[[m]] <- complete(imp2025, action = m)
}
for(m in 1:20){
  data.imputed[[m]] <- cbind(data.imputed[[m]], dataformerging)
}

# get average imputed values for prediction markers
prediction_marker<-c(colnames(dataforimp[,c(2:9)]))
data.imputed_mean <- data.imputed[[1]][,c(1,15,11)]
for (o in 1:length(prediction_marker)) {
  average_marker <- matrix(0, nrow(data.imputed[[1]]))
  for (m in 1:length(data.imputed)) {
    average_marker <- average_marker + data.imputed[[m]][,o+1]
  }
  average_marker <- average_marker / length(data.imputed)
  data.imputed_mean <- cbind(data.imputed_mean,average_marker)
  colnames(data.imputed_mean)[o+3] <- paste(prediction_marker[o])
}
prediction_marker_age<-c(colnames(data.imputed[[1]][,c(2:9)]),"Age")
```

3) a random forest prediction model was trained using the imputed data bundle (with 15,900 data entries) to predict the DPI based on predictors mentioned in Supplementary Table S1 (dpi ~ CD19 + CD20 + CD28 + CD45 + CD56 + CD138 + HLA.DR + Ki67). Only data entries corresponding to the day post immune intervention (dpi) of 5 and later were included to train the model solely on data from ongoing immune reactions.

```r
# bundle the imputed data for training the prediction model
data.full <- list()
for(m in 1:iter){
  data.full[[m]] <- complete(imp2025, action = m) %>%
    left_join(dataformerging, by = c("sample")) %>%
    mutate(group = as.factor(group)) %>%
    filter(dpi>4) #apply cut-off for dpi
}

# define a function to make and average predictions
average_predictions <- function(data, models) {
  # apply predictions using each model in the list
  predictions_list <- map(models, ~ predict(.x, newdata = data))

  # bind all predictions into a dataframe
```

```
  predictions_df <- bind_cols(predictions_list)

  # calculate the row-wise average of all predictions
  predictions_df <- predictions_df %>%
    mutate(p = rowMeans(across(everything()))) %>%
    mutate(ID = data$sample,dpi = data$dpi) %>%
    select(ID,dpi,p)

  return(predictions_df)
}

# train the random forest model
set.seed(557)
rf_ascmi <- data.full %>%
  map(~
        randomForest(dpi ~ CD19+CD20+CD28+CD45+CD56+CD138+HLA.DR+Ki67,
                     data = .,
                     ntree = 100,
                     mtry = 6,
                     importance = TRUE,
                     proximity =TRUE
        ))

# extract importance of predictors
df_list <- rf_ascmi %>%  map(~.$importance)
df_list <- lapply(df_list, as.data.frame)
add_id_variable <- function(df) {
  df <- df %>%
    mutate(ID = row.names(.))  # Create "ID" variable with row names
  return(df)
}
df_list <- lapply(df_list, add_id_variable)
variable_importance <- df_list %>%
  bind_rows() %>%
  group_by(ID) %>%
  summarise_all(mean, na.rm = TRUE)
```

The trained model candidate was applied to the training data to evaluate the model performance.

```
# use model on imputed data and average out predictions
prediction_all <- list()
for (m in 1:length(data.imputed)) {
  prediction_all[[m]] <- average_predictions(data.imputed[[m]], rf_ascmi)
}
mean_predictions <- matrix(0, nrow(prediction_all[[1]]), ncol(prediction_all[[1]])
)
for (m in 1:length(prediction_all)) {
  mean_predictions <- mean_predictions + prediction_all[[m]][,3]
}
mean_predictions <- mean_predictions / length(prediction_all)
predictions_mean <- cbind(dataformerging,dataforimp[,c(13,14,12)], prediction_all[
[1]][, 1:2], mean_predictions)
# trim output table
model_output<-predictions_mean %>%
  select(sample, StudyID,SubjectID, organ, group, dpi, p) %>%
```

```
  mutate(Age=dataforimp$Age) %>%
  mutate(Gender=dataforimp$Gender) %>%
  filter(dpi>4)
model_output$Age<-as.numeric(model_output$Age)

# correlate actual and predicted dpi using pearson correlation
r_HC_kinetic<-cor.test(model_output$dpi, model_output$p)
plot_HC_kinetic<-ggplot(model_output, aes(x=dpi, y=p)) +
  geom_point(shape=1) + #none-filled circles
  geom_smooth(method = "lm", color = "red", se = TRUE , level = 0.99) + #add regre
ssion line /w confidence interval
  labs(title = paste("pearson correlation, r =",format(r_HC_kinetic$estimate, digi
ts=3)," p =",format(r_HC_kinetic$p.value, digits=3)),
       x = "actual DPI",
       y = "predicted DPI") +
  theme_bw() +
  theme(legend.position = "none",
        axis.text.y = element_text(size = 20,face = "bold", colour = "black"),
        axis.title.y = element_text(size = 20,face = "bold"),
        axis.text.x = element_text(size = 20,face = "bold", colour = "black"),
        axis.title.x = element_text(size = 20,face = "bold"),
        plot.title = element_text(size = 15,face = "bold"))
print(plot_HC_kinetic)
```

4) several models using various predictor combinations were trained and evaluated based on the r-value of a Pearson correlation with actual DPI and predicted DPI. Four predictors (CD44, BCL-2, BCMA, and TACI) were directly omitted due to their high percentage of missing data and absence in the validation dataset. Age, sex, CD69, and CD86 were tested in various alterations of the prediction model but lacked added value. Additionally, values for parameters like ntree=100|200|300|500 and mtry=3|4|5|6|7 were tested and evaluated within the model development

5) a final random forest model using eight predictors mentioned above, with the final setting of ntree=100 and mtry=6 was chosen.

### *Application of the random forest prediction model to a new dataset*

As an example how the prediction model is further applied to new data, we describe here the steps used for validation of the ASC-ME model. Other input data in the right formatting can be processed using the same workflow. Alternatively, correctly formatted data can be uploaded to the accompanying online tool available via https://steinmetz.shinyapps.io/asc_me/

```
# define the data input for applying the ASC-ME model
data_new <- val_df %>%
  select(-c(CD98,Bcl.2,BAFFR,BCMA,TACI,CD44,CD95,CD69,CD86,subfolder,V2,Race,Race.
Specify,Ethnicity,ARM.Accession,Expsample.Accession,Original.File.Name)) %>%
  filter(!(sample=="NA")) %>%
  rename(dpi = Study.Time.Collected) %>%
  rename(group = ARM.Name) %>%
  rename(datatype = File.Detail) %>%
  rename(Age = Subject.Age) %>%
  rename(StudyID = Study.Accession) %>%
  rename(SubjectID = Subject.Accession) %>%
  rename(organ = Biosample.Type) %>%
  mutate(across(3:10, ~ ifelse(.x < -2 | .x > 5, NA, .x)))
```

```
new_merging <- data_new %>%
  select(sample,count,source_file,SubjectID)
new_imp <- data_new %>%
  select(-c(count,source_file,SubjectID))

# the new data is combined with the imputed training data ensuring that small data
sets are less subject to
# imputation bias.
iter=20
data.norm<-cbind(data.imputed_mean[,c(1,4:11)],dataforimp[,c(10:16)])
data.total<-rbind(data.norm,new_imp)
imp.new <- mice(data.total, method = "rf", m = iter, seed = 557)

# bind imputed new data into a list and add metadata excluded from imputation
new.imputed <- list()
for(m in 1:20){
  new.imputed[[m]] <- complete(imp.new, action = m)
}
for(m in 1:20){
  new.imputed[[m]] <- cbind(new.imputed[[m]][c((nrow(data.norm)+1):nrow(new.impute
d[[m]])),], new_merging)
}

# use model on all imputed new data
prediction_new <- list()
for (m in 1:length(new.imputed)) {
  prediction_new[[m]] <- average_predictions(new.imputed[[m]], rf_ascmi)
}

# calculate the average prediction and trim the output table
new_predictions <- matrix(0, nrow(prediction_new[[1]]), ncol(prediction_new[[1]]))
for (m in 1:length(prediction_new)) {
  new_predictions <- new_predictions + prediction_new[[m]][,3]
}
new_predictions <- new_predictions / length(prediction_new)
predictions_new <- cbind(new_merging,new_imp[,c(13,14,12)], prediction_new[[1]][,
1:2], new_predictions)

new_output<-predictions_new %>%
  select(sample, SubjectID, group, dpi, p) %>%
  mutate(Age=new_imp$Age) %>%
  mutate(Gender=new_imp$Gender)
new_output$Age<-as.numeric(new_output$Age)

val_data<-new_output %>%
  filter(dpi>4)

# correlate actual and predicted dpi for the new data using pearson correlation he
re for validation purposes
r_ex_kinetic<-cor.test(val_data$dpi, val_data$p, method = "pearson")
val_plot_scatter<-ggplot(val_data, aes(x=dpi, y=p)) +
  geom_point(shape=1) + #none-filled circles
  geom_smooth(method = "lm", color = "red", se = TRUE ) + #add regression line /w
confidence interval
  labs(title = paste("pearson r =",format(r_ex_kinetic$estimate, digits=3)," p =",
```

```
format(r_ex_kinetic$p.value, digits=3)),
       x = "actual DPI",
       y = "predicted DPI") +
  theme_bw()+
  theme(legend.position = "none",
        axis.text.y = element_text(size = 20,face = "bold", colour = "black"),
        axis.title.y = element_text(size = 20,face = "bold"),
        axis.text.x = element_text(size = 20,face = "bold", colour = "black"),
        axis.title.x = element_text(size = 20,face = "bold"),
        plot.title = element_text(size = 15,face = "bold"))
print(val_plot_scatter)
```
